# Visualization of T Cell Migration in the Spleen Reveals a Network of Perivascular Pathways that Guide Entry into T Zones

**DOI:** 10.1016/j.immuni.2020.03.010

**Published:** 2020-05-19

**Authors:** Anne Chauveau, Gabriela Pirgova, Hung-Wei Cheng, Angelina De Martin, Felix Y. Zhou, Sarah Wideman, Jens Rittscher, Burkhard Ludewig, Tal I. Arnon

**Affiliations:** 1 University of Oxford, Kennedy Institute of Rheumatology, OX3 7FY Oxford, UK; 2 Institute of Immunobiology, Kantonsspital St. Gallen, 9007 St. Gallen, Switzerland; 3 University of Oxford, Ludwig Institute for Cancer Research, OX3 7DQ Oxford, UK; 4 University of Oxford, Institute of Biomedical Engineering, OX3 7DQ Oxford, UK; 5 University of Oxford, Big Data Institute, Li Ka Shing Centre for Health Information and Discovery, OX3 7XP Oxford, UK

**Keywords:** intravital imaging, spleen, T cells, lymphocyte trafficking, bridging channels, vascular-guided migration, GPCRs

## Abstract

Lymphocyte homeostasis and immune surveillance require that T and B cells continuously recirculate between secondary lymphoid organs. Here, we used intravital microscopy to define lymphocyte trafficking routes within the spleen, an environment of open blood circulation and shear forces unlike other lymphoid organs. Upon release from arterioles into the red pulp sinuses, T cells latched onto perivascular stromal cells in a manner that was independent of the chemokine receptor CCR7 but sensitive to Gi protein-coupled receptor inhibitors. This latching sheltered T cells from blood flow and enabled unidirectional migration to the bridging channels and then to T zones, entry into which required CCR7. Inflammatory responses modified the chemotactic cues along the perivascular homing paths, leading to rapid block of entry. Our findings reveal a role for vascular structures in lymphocyte recirculation through the spleen, indicating the existence of separate entry and exit routes and that of a checkpoint located at the gate to the T zone.

## Highlights

• Perivascular pathways support T cell entry into splenic T zones, but not egress from them
• Attachment to the homing paths requires activation of GPCRs other than CCR7
• CCR7 mediates one-directional migration and entry into T zones
• Inflammation leads to modification of the homing paths and to rapid block of entry

## Graphical Abstract

**Figure:**
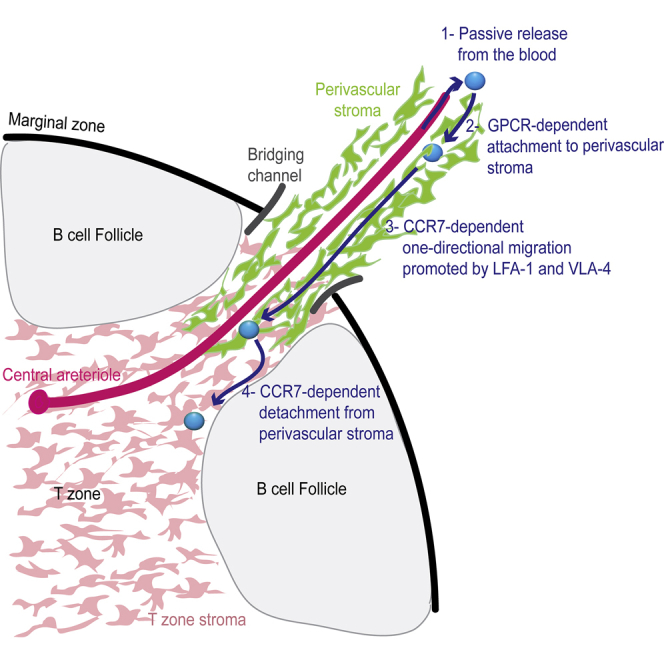

## Teaser

Lymphocyte recirculation between secondary lymphoid organs is critical for their function and for immune homeostasis. Using intravital imaging, Chauveau et al. reveal that a network of perivascular pathways supports T cell migration into splenic T zones and describe a dynamic multi-step cascade of entry.

## Introduction

Adaptive immunity depends on the ability of lymphocytes to circulate between secondary lymphoid organs. This process is critical for immune surveillance, ensuring effective activation of rare antigen-specific cells, as well as during homeostasis, where it provides cells with regular access to survival and peripheral tolerance-promoting signals (Butcher and Picker, 1996, Cyster, 1999). In lymph nodes (LNs) and other secondary lymphoid organs, specialized post-capillary venules known as high endothelial venules (HEVs) provide the entry sites into the tissue, where a well-defined cascade of events leading to cell entry has been described (Butcher and Picker, 1996, Girard et al., 2012, Luster et al., 2005). These studies advanced our basic understanding of the immune system and have led to the development of approved agents that inhibit leukocyte entry as treatments for immunological diseases (Kaneider et al., 2006, Ozerlat, 2011). However, in the spleen, HEVs are absent and the mechanisms that regulate cell trafficking through this organ are poorly defined (Lewis et al., 2019, Mebius and Kraal, 2005, Tadayon et al., 2019). Given that more cells pass through the spleen than via all other secondary lymphoid organs combined (Ford and Gowans, 1969), the contribution of this organ to cell recirculation is fundamental. Without delineating the mechanisms involved, our understanding of the overall process remains limited.

Lymphocytes enter the spleen with the blood flow and are first released into the red pulp and marginal zone (MZ) compartments, where central arterioles open and release their blood content (Lewis et al., 2019, Mebius and Kraal, 2005, Tadayon et al., 2019). From there, T cells migrate into the lymphoid compartment of the white pulp in an active and regulated process (Cyster and Goodnow, 1995) that requires the chemokine receptor CCR7 (Förster et al., 1999). Yet, the path taken and mechanisms that mediate the transition into the white pulp are poorly understood. Early histological studies identified sites known as the MZ bridging channels (BCs) as ports of entry into the T zone (Brelińska et al., 1984, Kotani et al., 1986, Mitchell, 1973, Nieuwenhuis and Ford, 1976). Confocal analysis of spleen sections further suggested that at these sites, a network of T zone fibroblastic reticular cells (TRCs) extends and connect to the T zone, possibly providing guidance to migrating cells (Bajenoff et al., 2008). Together, these studies led to a model suggesting that upon arrival to the spleen, T cells either “drift” with the blood flow or migrate freely in the blood-exposed compartments until reaching a BC, where open channels in the MZ may allow direct contact with extensions of TRC networks and facilitate passage into the T zone. However, how newly arriving cells that are initially released in the red pulp and MZ are able to maintain directional migration toward a BC, despite the presence of shear forces that limit cell movement in this compartment (Arnon et al., 2013), remains unclear. It has also been difficult to reconcile the proposed existence of open “breaks” in the MZ shell that are thought to be deprived of physical structures with the notion that blood and other substances in the circulatory fluids do not “leak” into the white pulp. Finally, it remains unknown whether the BCs permit bidirectional migration, as was originally proposed (Mitchell, 1973), or whether instead entry and egress in the spleen are mediated via different non-overlapping structures.

In recent years, advances in intravital imaging approaches have greatly improved our ability to analyze dynamic cell behavior deep within splenic compartments (Arnon et al., 2013). Here, we applied this approach to investigate the pathways and mechanisms used by naive T cells during entry into splenic T zones. We found that circulating T cells migrated along a network of stromal-cell-coated perivascular pathways, which supported entry into T zones, but not egress from them. CCR7 was crucial for one-directional migration along these pathways and for entry into the T zone compartment; however, another Gi protein-coupled receptor(s) (GPCR), in addition to CCR7, was necessary for initial attachment to them. Finally, we found that during inflammation, the homing pathways were rapidly modified, leading to a block in entry. Our study sheds light on one of the key steps in lymphocyte recirculation and reveals a role for the vasculature system in supporting cell migration within the spleen.

## Results

### T Cell Movement in the Marginal Zone and Red Pulp Is Limited

Blood containing circulating lymphocytes initially enters the spleen via central arterioles that run through the T zones. Branches extending from these then release their blood cargo into the MZ and red pulp via terminal branching vessels (Figures S1A–S1C). However, standard histology suggests that only a fraction of these vessels end in close proximity to a BC and that many newly arriving T cells are initially released in more distal locations in the red pulp compartment (Tadayon et al., 2019). Since previous studies have not identified routes for T cells passing outside the T zone, it is unclear how newly arriving cells migrate within these regions to access a remote entry site. To address this directly, we first analyzed T cell behavior in the MZs and red pulps of live mice. To highlight T cells, we used hCD2*-DsRed* transgenic mice, where the fluorescent protein DsRed is expressed under the human CD2 (*hCD2*) promotor. The follicular compartments were demarcated by transferring large numbers of B cells expressing enhanced green fluorescent protein (GFP) under the human *UBC* promoter (hUbi*-GFP*). Spleens of anesthetized mice were surgically exposed and imaged (Arnon et al., 2013). The approximate location of the MZ was defined as the area that immediately interfaces with the follicles, as previously described (Arnon et al., 2013).

**Figure 1:**
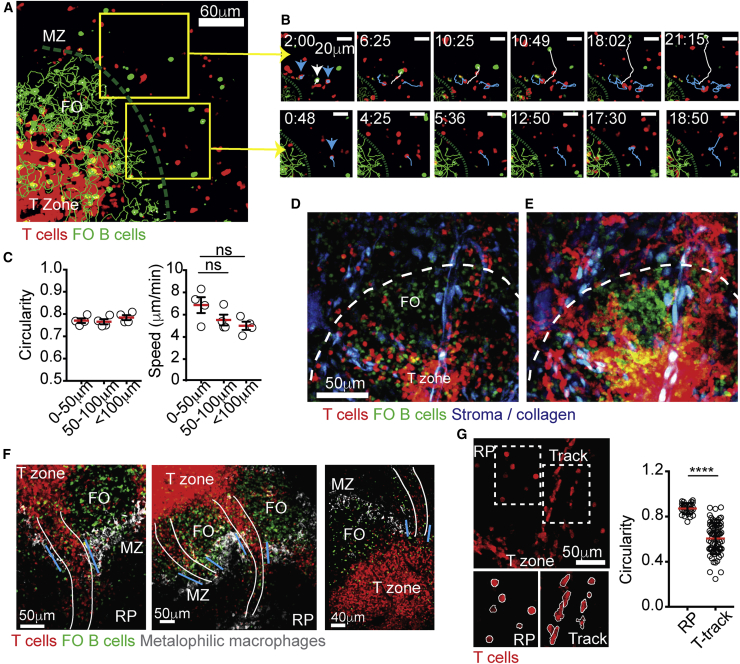
T Cells Migrate along Tracks that Extend between the T Zone and Red Pulp Compartments (A) Two-photon laser scanning microscopy (TPLSM) of spleens from hCD2*-DsRed* mice (endogenous T cells, red) transferred with GFP^+^ B cells (green). Green line indicates the approximate location of the border between the marginal zone (MZ) and follicles (FO). (B) Closer view of two areas in the MZ region (yellow boxes in A). The movement of T cells (blue arrow and line) and B cells (white arrow and line) over time is highlighted. See also Video S1. (C) Circularity index and velocity of endogenous T cells migrating in the MZ and red pulp (RP). The x axis represents distance of cells from the MZ-FO border. Each circle represents the average circularity or velocity of cells assessed in one mouse. Error bars represent SEM. Data in (A–C) represent at least four independent experiments. (D and E) TPLSM of Actin-*CFP* chimeras reconstituted with a 1:1 mixture of bone marrow from CD19^cre−cre/*YFP*^ (follicular B cells, green) and hCD2*-DsRed* (T cells, red) mice. Collagen (detected with second harmonic) and CFP signals were collected in the same detector (blue). 69-μm z-projection (D) and time projection (E) views are shown. The dashed white line indicates approximate location of the MZ-FO border. See also Video S2. (F) Histological analysis of fixed spleen sections of hCD2*-DsRed* mice (endogenous T cells, red) adoptively transferred with GFP^+^ B cells (green) and stained for CD169 (gray). Images were taken 24 h after B cell transfer. Tracks of T cells are highlighted with a white line. Blue lines indicate location of MZ BCs. (G) Left, a snapshot of a 2D slice from a TPLSM sequence illustrating cell shape. Bottom, examples of masks drawn around T cells to calculate circularity. Right, circularity index of T cells migrating in the red-pulp (RP) and on T cell tracks (“T-track”). Error bars represent SD. Data in (D–G) represent at least three independent experiments. ^∗∗∗∗^p < 0.0001.

In contrast to T cells in the T zone that migrated extensively during imaging periods, T cells within the MZ and red pulp were mostly rounded and immotile, with some being caught in regions of blood flow as indicated by their displaying sharp linear movements toward or within the red pulp (Figures 1A–1C; Video S1). This behavior is consistent with the way by which follicular B cells were reported to move in this region (Arnon et al., 2013). Despite this, however, a second behavior by smaller frequencies of T cells was also observed in the same areas (Figure 1B, top panel). Consequently, the average velocity of T cells that were located more proximal to the follicles was slightly higher than the speed of T cells located further in the red pulp (Figure 1C). This effect might partially be explained by higher levels of lymphocyte function-associated antigen 1 (LFA-1) expression on T cells compared to follicular B cells (Figure S1D), in a manner similar to the role of integrins in mediating retention and migration of MZ B cells in this compartment (Arnon et al., 2013, Lu and Cyster, 2002). In the red pulp, T cells were almost completely stationary, demonstrating poor displacement and low velocities (Figure 1C; see also 2B). The lower levels of ICAM-1 expression in the red pulp compared with the MZ (Lu and Cyster, 2002) may partially explain the greater reduction in motility in this compartment. These results demonstrate that similarly to follicular B cells, the ability of circulating T cells to actively migrate in the red pulp and MZ is limited, calling into question the central concept that T cells freely navigate within these compartments in search of remote BCs.

**Figure 2:**
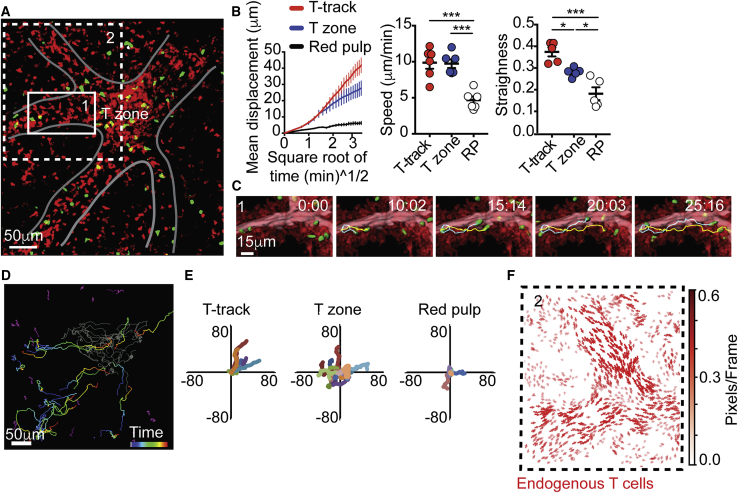
T Cells Move in a One-Directional Manner toward T Zones (A) 87-μm z-projection view showing endogenous hCD2*-DsRed* T cell (red) and transferred GFP^+^ T cells (green) imaged 24 h after transfer. Grey line highlights tracks of T cells connected to a T zone. Boxes 1 and 2, closeup of areas shown in (C) and (F), respectively. See also Video S4. (B) Mean displacement versus square root of time (left), mean velocities (middle), and straightness of migration path of transferred GFP^+^ T cells (right) in the indicated regions. Data are representative of 5 independent mice. Each dot represents the mean of speed or straightness of at least 40 cells analyzed in each mouse. Lines in the left graph represent the average mean displacement calculated in five mice analyzed. Error bars represent SEM. (C) Time-lapse series of transferred T cell movement toward a T zone. Image shows two representative examples of T cell trajectories (yellow and cyan lines) overlaid on surfaces created based on time projection of endogenous T cells (red). In pink, the lumen of the T-tracks is highlighted using a manually drawn surface. (D) Representative classification of T cell trajectories in the T zone (gray), red pulp (purple), and tracks of T cells (“T-tracks,” time color mapped). (E) Superimposed 10 min of randomly selected T cell trajectories in the indicated compartments. (F) Cell motion pattern of endogenous T cells (red) (closeup of area shown in A, Box 2) using motion sensing superpixels (MOSES) analysis. Arrows represent direction of displacement. Arrow length represents the velocity of superpixel displacement (see STAR methods). Data in (A–F) represent at least five independent experiments. ^∗∗∗^p < 0.001; ^∗^p < 0.05.

Video S1. Intravital Imaging of T Cells and B Cells in the MZ, Related to Figure 1 Time-lapse series from intravital TPLSM (87-μm-thick z stack) of hCD2-*DsRed* spleens 24 h after transfer with GFP^+^ B cells (green). The video starts with an overview of the imaged area showing the T zone (identified by high concentration of T cells) and B cell FO, identified and marked by high density of B cell tracks (green). The border between the compartments is indicated with a white line. Two regions in close proximity to the MZ are marked as box 1 and 2 and are zoomed on in the following two sequences. Zoom 1 shows a typical example of B cell behavior in the MZ; in this region, B cells lost their migratory capacity and alternated between stationary and fast movement (white line). In contrast, 2 examples of T cells that continue to actively migrate in the same area are highlighted (blue line). Zoom 2 shows an example of a T cell, which similarly to B cells, lost its ability to migrate and displayed fast directional path movement away from the follicles. Elapsed time is shown as min:sec. Data are representative of at least five independent experiments.

To first identify paths of T cell entry into the white pulp, we generated bone marrow (BM) chimeric mice in which the various splenic compartments could be identified more readily, without the need for transfer of a large number of B cells. To this end, mice were reconstituted with a mixture of hCD2*-DsRed* and CD19*-cre* Rosa26*-eYFP* (CD19^cre−cre/*YFP*^) BM cells, such that ∼50% of the T cells were red (expressing DsRed) and ∼50% of the follicular B cells were yellow (expressing YFP). Since the Cre interrupted both *Cd19* alleles, MZ B cells that require CD19 to develop (Martin and Kearney, 2000) arose from hCD2*-DsRed* donors but remained invisible, allowing selective labeling of the follicular compartment. As such, the follicular compartment was selectively labeled as being YFP positive and T cells as DsRed positive in these mice. Transgenic mice expressing cyan fluorescent protein under the *Actin* promotor (Actin-*CFP*) were used as recipients in order to provide additional structural information from the non-hematopoietic cells. This approach allowed us to frequently locate regions in which B cell follicles were adjacent to T zones (Figure 1D; Video S2). To obtain a general overview of cell motility and identify regions of extensive cell movement, videos were projected over time (Figure 1E). Whereas most T cells were confined to the T zone, a sizable population accumulated on “track-like” structures that appeared to connect the MZ and T zone compartments (Figures 1D–1F). T cells associated with tracks had a more elongated morphology than cells in the red pulp, consistent with higher motility (Figure 1G).

Video S2. Intravital TPLSM of T Cell and Follicular B Cell Migration in the Splenic White Pulp, Related to Figure 1 Time-lapse image sequence (69-μm-thick z stack) from intravital TPLSM of T cells (red) and follicular B cells (green) migrating in the spleen of CFP-actin recipient chimeras reconstituted with a 1:1 mixture of bone marrow from CD19^*cre/cre/YFP+*^ and hCD2-*DsRed* mice. Stromal cell (expressing CFP) and collagen (second harmonic signal) are detected in the same detector and shown in blue. At the start of the video, the main compartments are annotated, and the MZ-FO border is highlighted with a white line. Arrows mark tracks of T cells. At the end of the sequence, a time projection of the video is displayed to emphasize areas of extensive cell movement. Elapsed time is shown as min:sec. Data are representative of at least five independent experiments.

Many of the tracks extended deep toward the red pulp, where circulating lymphocytes were recently shown to be frequently released from the blood (Tadayon et al., 2019). Further examination of the position of the tracks relative to other compartments suggested that these paths connected the white pulp and red pulp by crossing a BC, where the MZ macrophage ring is “broken” and where a gap between two neighboring follicular B cell compartments allow uninterrupted access to a T zone (Figure 1F). Some tracks were found to travel above B cells zones, but they did not pass directly inside them (Figure S1E; Video S3). Thus, while the BCs have been previously described as “open doors” that form direct connection between compartments, our findings suggest that these portals are more complex and that distinct paths that support T cell migration pass through them. Because live imaging analysis allowed preservation of these pathways, which largely collapsed during processing of tissue sections, we were able to directly identify and define their cellular composition and function.

Video S3. Visualization of T Cell Migration Tracks in Relation to B Follicles and a T Zone, Related to Figure 1 3D representation (69-μm-thick z stack) of a spleen from intravital TPLSM showing GFP^+^ B cells (green) 24 h after transfer into hCD2-*DsRed* mouse (endogenous T cells, red). This 3D overview shows the architectural organization of the bridging channels relative to B cell follicles (FO) and a T zone. The T cell tracks are identified by accumulation of migrating T cells and are highlighted by a second harmonic signal of collagen fibers (blue). Tracks of T cells are seen passing in close proximity and above FO compartments before reaching the top of the T zone. From there, the cells “dive” into the T zone compartment. The video relates to the images shown in Figure S1E.

### Naive Circulating T Cells Use Distinct Sites for Entry and Egress

In LNs, specialized non-overlapping structures have evolved to support entry and egress, with T cells entering from the blood via HEVs and leaving into the efferent lymphatic by crossing the cortical and medullary sinuses (Girard et al., 2012, von Andrian and Mempel, 2003). However, in the spleen, the anatomy of cell migration is less clear, and it is unknown whether entry and egress are mediated via the same or distinct sites (Khanna et al., 2007, Mitchell, 1973). To address this question, we quantitatively assessed the behavior of GFP^+^ T cells that have been transferred into hCD2*-DsRed* mice 24 h prior to imaging (Figures 2A–2F; Video S4). The trajectories of transferred T cells were manually annotated and the migratory parameters of cells in the different compartments assessed. Track-associated T cells displayed high motility and moved with velocities that were similar to those measured in T zones (Figure 2B). However, when on tracks, the cells traveled in a straighter manner and displaced larger distances, reflecting a highly directional migration (Figures 2B and 2C). Over 82% ± 5.8% of track-associated T cells were moving in a single direction, migrating from the red pulp and MZ toward the T zone (Figures 2D and 2E; Video S4). This was also the case when cells were transferred for shorter periods of time (4–8 h, Figures S2A–S2D). Often, cells could be seen migrating on the tracks and ending deep within T zones (Video S4). Endogenous T cell trajectories extracted by motion sensing superpixels (MOSES) analysis (Zhou et al., 2019) showed similar one-directional movement (Figure 2F). This behavior was consistent among more than 12 mice and 24 videos analyzed. In contrast, we could not find similar structures that supported migration of transferred T cells in a one-directional manner away from T zones toward the red pulp or MZ compartments even 3 days post-transfer, at a time when egress events should be frequent (Figures S2E and S2F). Furthermore, despite extensive efforts, no collective egress movement of endogenous T cells could be identified. These results provide direct evidence to the notion that entry and egress from the splenic white pulp are mediated via distinct non-overlapping sites, indicating the existence of a separate exit route.

Video S4. T Cell Tracks Facilitate One-Directional Migration of T Cells Deep into the Splenic T Zone, Related to Figure 2 Two examples of time-lapse series from intravital TPLSM (87-μm-thick z stack) of spleen, demonstrating the migration of GFP^+^ T cells (green) 24 h after transfer into hCD2-*DsRed* mouse (endogenous T cells, red). The video starts with an overview of the imaged area highlighting multiple T cell tracks leading into a T zone (white line). Most of the transferred T cells can be seen migrating on the tracks and accumulating inside the T zone over time. A zoomed-in view from this time sequence is then displayed and processed to better highlight the structure of the tracks. Time projection of endogenous T cells (red) was generated, and automated surface was created around it to highlight areas of extensive cell movement. Two T cell tracks can be seen converging into the beginning of a T zone. A manually drawn surface was created using the DsRed signal to visualize the lumen of the T cell track (pink). Transferred T cells are shown with surface seed points created with Imaris (green). T cells often moved in a highly directional and straight manner along the tracks toward T zones. Movement can be seen outside of the lumen, on the outer layer of the tracks. Two examples of T cell trajectories are shown. In the second example, the video is displayed using horizontal (XY) and orthogonal (XZ) projections of z stacks to illustrate the relative positioning of the T zone bellow the channel, and to show examples of cells entering the T zone by diving deep into this compartment. An example of two trajectories of migrating T cells are shown (time colored). Elapsed time is shown as min:sec. Data are representative of at least eight independent experiments.

### T Cells Use Vascular Structures to Guide Their Migration into T Zones

**Figure 3:**
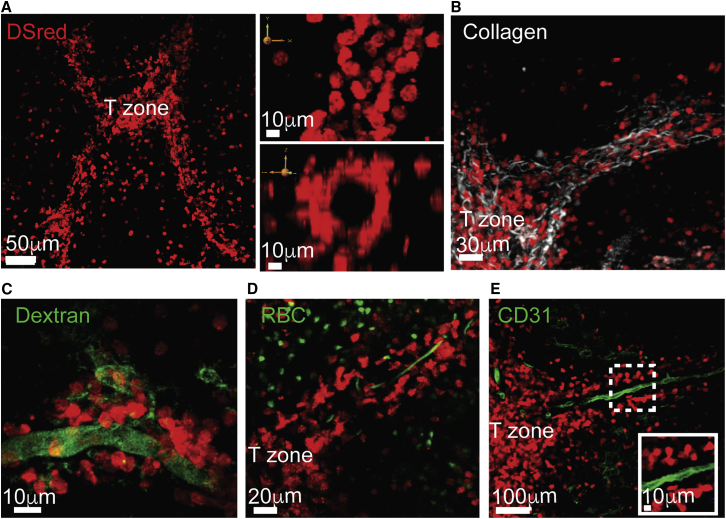
Homing Paths of T Cells Are Guided by Blood Vessels (A–D) Snapshots from intravital imaging of live hCD2-*DsRed* spleens by TPLSM. (A) Left, 3D view of a 70-μm z-projection. Right panels, XY (top) and ZY (bottom) are views of a magnified part of the same area. (B) 105-μm z-projection of endogenous T cell (red) and collagen (second harmonic, white). Data in (A) and (B) are representative of at least 20 mice and 40 videos recorded. (C) 3D view of 27-μm z-projection of a spleen a few minutes after intravenous injection of Dextran-FITC 2,000 kDa (green). Data are representative of at least three mice and six videos acquired. See also Video S5. (D) 18-μm z-projection of GFP^+^ red blood cells (RBC, green) injected into a hCD2*-DsRed* host. Data are representative of at least six mice and 14 videos acquired. See also Video S6. (E) Fixed spleens from hCD2*-DsRed* mice were sliced with a vibratome (150 μm thick) and stained for CD31. Micrographs show high magnifications of areas highlighted in dotted box. Sections were imaged by TPLSM. Data represent sections from at least three mice analyzed.

The identification of these homing routes in the spleen prompted us to further explore their cellular composition and structural organization. Using live imaging, we noted that the T cell tracks were associated with large collagen bundles and appeared hollow in the middle, forming a cylinder-like shape (Figures 3A and 3B). To test whether they may be part of the vasculature or conduit systems, we intravenously injected hCD2*-DsRed* mice with a high molecular weight fluorescein isothiocyanate (FITC)-dextran (2,000 kDa). *In vivo* imaging showed rapid labeling of the luminal side of the tracks (Figure 3C; Video S5), supporting the possibility of them being blood vessels (Nolte et al., 2003). To directly test this hypothesis, we visualized blood flow by injecting GFP^+^ red blood cells into hCD2*-DsRed* recipients shortly before imaging. By limiting our imaging volumes to thin layers (15–35 μm), we were able to increase the rate of image acquisition, such that rapid events could be captured. Indeed, many GFP^+^ cells were observed to move at very high speeds, often appearing as “flying” cells in the center of the entry tracks, confirming the presence of blood flow (Figure 3D; Video S6). However, while every T-cell-associated track that we could identify showed evidence for blood flow, not all the blood vessels in the spleen were associated with T cells, which highlighted the specialized nature of the homing tracks (Figures S3A–S3C). To further explore their cellular composition and association with blood vessels, we optimized an approach for visualizing them *ex vivo*. Spleens were fixed and gently cut using a vibratome; sliced sections were then stained and imaged using two-photon laser scanning microscopy (TPLSM) (see STAR Methods). Although many T cell tracks collapsed during processing, some were preserved and could be imaged. A distinct stain of CD31^+^ cells was observed within the inner layer of the tracks, consistent with the presence of blood endothelial cells (Figure 3E).

Video S5. Intravital TPLSM of the Spleen after Intravenous Injection of High Molecular Weight Dextran-FITC, Related to Figure 3 Time-lapse series from intravital TPLSM (27-μm-thick z stack) of endogenous T cells (hCD2-*DsRed*, red) in a live spleen few minutes after intravenous injection of Dextran-FITC 2,000 kDa (green). The video focuses on a T cell homing path passing via the red pulp, showing tracks covered with migrating T cells becoming rapidly labeled. Elapsed time is shown as min:sec. Data are representative of at least three mice and six videod acquired.

Video S6. The Homing T Cell Tracks Are Guided by Blood Vessels, Related to Figure 3 Three examples of intravital imaging of the spleen (18-μm z stack, example 1; 36-μm z stack, example 2; 60-μm z stack, example 3) of a hCD2-*DsRed* mouse injected with GFP^+^ red blood cells (RBC, green). Videos were taken minutes after RBC injection. The first example is focused on an area near a T zone, the second on an area further away toward the red pulp, and the last one on an area containing multiple PT-tracks. RBCs can be seen moving in very high speeds in the middle of the T cell tracks, indicating blood flow. Elapsed time is shown as min:sec. Data are representative of at least six mice and 14 videos acquired.

Taken together, these results demonstrated that the homing paths of T cells are structurally coupled to blood vessels, creating perivascular scaffolds upon which cells migrated in a highly directional manner toward T zones. By attaching to and moving along the outside of blood vessels that release newly arriving lymphocytes into the red pulp, T cells were able to “backtrack” their steps and be efficiently guided to T zone entry sites. This structural organization highlights an unconventional role for the vasculature system in the spleen, supporting cellular migration not only between organs, but also across distanced compartments within the same tissue. We propose to refer to these paths as “perivascular T-tracks” (PT-tracks, in short), to distinguish them from other types of cellular pathways described in the spleen.

### Perivascular T-Tracks Are Coated with a Unique Subset of Reticular Cells

**Figure 4:**
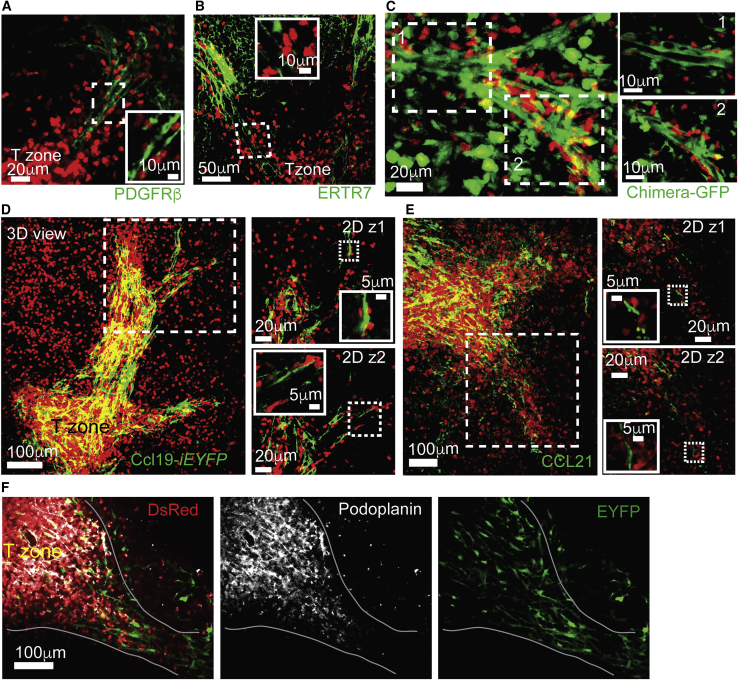
T Cells Migrate on Perivascular Stromal Cells Connecting the Red Pulp to T Zones (A and B) Fixed spleens from hCD2*-DsRed* mice were sliced with a vibratome (150 μm thick) and stained for PDGFR-β (A) or ER-TR7 (B). Micrographs show high magnifications of areas highlighted in dotted boxes. Sections were imaged by TPLSM. (C) Left, snapshot from intravital imaging of spleens from a hUbi-*GFP* (green, stroma) recipient chimera reconstituted with hCD2*-DsRed* bone marrow cells (red, T cells). Right, zoom on two boxed regions showing a single z-plane. (D) Sections from Ccl19*-iEYFP.2* chimeras reconstituted with hCD2*-DsRed* bone marrow stained with anti-GFP (green). Two single z-planes from the indicated boxed region are shown on the right with additional micrographs for further magnifications of marked areas (dotted box). (E) Fixed spleens from hCD2*-DsRed* mice were vibratome-sliced (150 μm thick) and stained for CCL21 (green). Two single z-planes from boxed region are shown on the right with additional micrographs for further magnifications of marked areas (dotted box). Data are representative of two mice. (F) Fixed sections from Ccl19*-iEYFP.2* chimeras reconstituted as in (D) were stained with anti-GFP (green) and podoplanin (white). Sections were imaged by confocal microscopy. Data in (D) and (F) represent three mice, with at least six sections per mouse.

While blood vessels formed the scaffold of the PT-tracks, T cells did not appear to directly migrate on the blood endothelia. Instead, we found T cells were migrating on the outer layer of the vessels, closely interacting with platelet-derived growth factor receptor beta (PDGFRβ) and ER-TR7-expressing cells (Figures 4A and 4B). To better understand the structural organization of the PT-tracks and explore the nature of the coating layer upon which T cells migrated, we generated chimeras in which hUbi-*GFP* hosts were reconstituted with BM derived from hCD2*-DsRed* mice. In these mice, the stromal compartment was more readily visible compared with Actin-*CFP* hosts such that complete stromal networks could be visualized. TPLSM analysis revealed a “labyrinth-like” organization of multiple coating layers surrounding the PT-tracks with T cells migrating directly on them (Figure 4C). To further highlight specific stromal cell subsets, we utilized a variant of the *Ccl19*-tTA transgenic mouse model (Ccl19*-iEYFP.2*) where EYFP is permanently induced in CCL19-expressing cells (Cheng et al., 2019). In this model, Cre recombinase expression is restricted to TRCs and perivascular reticular cells (PRCs) (Figure S4A). The chemokines that mediate naive T cell recruitment to the splenic white pulp, i.e., CCL19 and CCL21a, were highly expressed in EYFP-positive fibroblasts in spleens of Ccl19*-iEYFP.2* mice (Figures S4B and S4C). To determine whether EYFP expression is associated with the PT-tracks, we reconstituted Ccl19*-iEYFP.2* mice with hCD2*-DsRed* BM. EYFP signal was detected in cells coating the PT-tracks, forming a continuous layer that expanded toward the T zone and eventually joined it (Figure 4D). In addition, staining with CCL21a antibody indicated that the chemokine was expressed along the PT-tracks (Figure 4E). However, in contrast to T zone stroma, PT-track-associated EYFP^+^ cells did not stain for podoplanin (gp38) (Figures 4F, S4D, and S4E). These results highlighted the cellular composition of the PT-tracks and suggested that both TRCs and PRCs form their structural basis and serve as a source of T-cell-attracting chemokines.

We observed that many PT-tracks often extended deep into the red pulp passing into blood-exposed regions (e.g., Video S6). As shown in Figures 1A–1C, in these areas, cell movement was dramatically reduced. Yet, T cells that were associated with PT-tracks migrated extensively (Figure 2). We hypothesize that the presence of a mesh of reticular cells surrounding these structures may provide a partial shield from blood flow, thus preventing T cells from being exposed to shear forces that limit migration. In agreement with this possibility, we found that in 5 min *in vivo* labeling (STAR Methods; Cinamon et al., 2008), accessibility of antibodies to PT-track-associated T cells within the red pulp regions was variable, with some T cells being rapidly labeled and others remaining unstained (Figure S4F).

### LFA-1 and VLA-4 Accelerate T Cell Migration along PT-Tracks

Integrins play critical roles in mediating cell adhesion and migration under shear stress (Sixt et al., 2006). In particular, LFA-1 and very late antigen-4 (VLA-4) are essential for T cell attachment to HEVs, and when blocked, cells quickly detach from the endothelial surface and are flushed away with the blood flow (Mempel et al., 2004). Similarly, retention and migration of MZ B cells in the blood-exposed MZ compartment of the spleen depend on interactions between LFA-1 and VLA-4 with their ligands ICAM-1 and VCAM-1, and when blocked, MZ B cells rapidly detach from the MZ and are displaced to the circulation (Arnon et al., 2013, Lu and Cyster, 2002). The contribution of integrins to homing of lymphocytes to the splenic white pulp has been unclear (Lo et al., 2003, Manevich-Mendelson et al., 2010, Nolte et al., 2002). We therefore considered whether integrins may play a similar role in the spleen as in LNs, supporting attachment of T cells to the PT-tracks.

**Figure 5:**
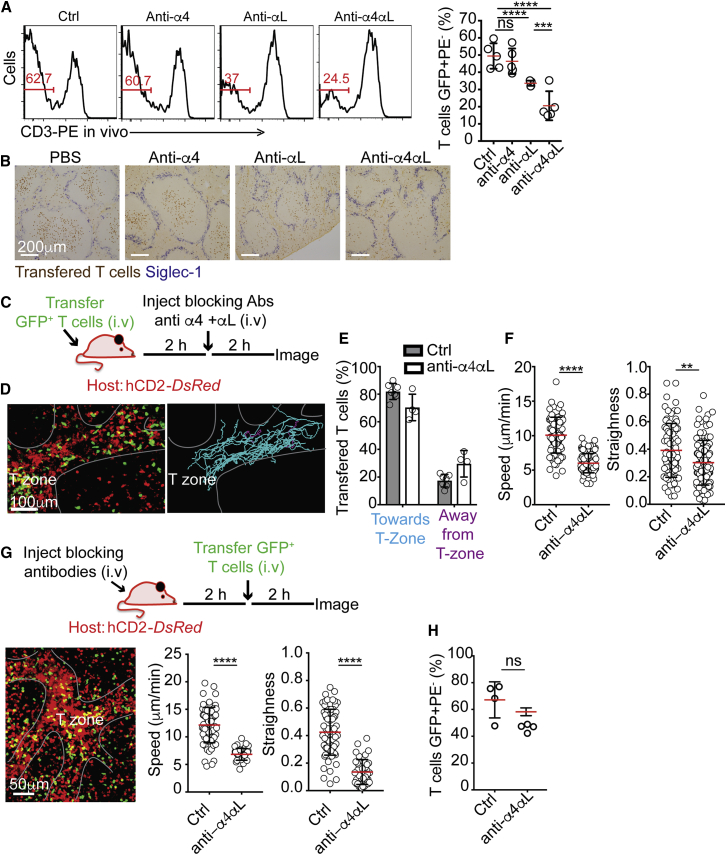
LFA-1 and VLA-4 Promote T Cell Migration during Entry (A) Flow-cytometric detection of GFP^+^ T cells labeled 5 min *in vivo* with anti-CD3-PE 2.5 h after being transferred into hosts injected with PBS (control) or blocking antibodies against integrin subunits α4, αL, or α4+αL, as indicated. Mice were treated with the above antibodies 2 h prior to T cell transfer. Red numbers show frequency of unlabeled cells. Right, summary showing one out of three representative experiments. Error bars represent SD. (B) Immunohistochemistry on spleen sections from mice treated as in (A), stained with anti-GFP (transferred T cells, brown) and anti-CD169 (to highlight the MZ macrophages, blue). Data are representative of four independent experiments. (C–F) TPLSM of hCD2*-DsRed* mice transferred with GFP^+^ T cells following combined (α4 and αL) integrin blockade. (C) Schematic of experimental design. (D) 63-μm z-projection view showing endogenous (red) and transferred (green) T cells. Grey line highlights PT-tracks connected to a T zone. Left, trajectories of transferred T cells with cyan lines indicating cell moving toward the T zone and purple lines showing cells moving away from it. (E) Summary of frequencies of cells migrating toward or away from the T zones. Each circle represents the average frequency measured in one mouse (n ≥ 4). (F) Mean velocities and straightness of migration path of transferred GFP^+^ T cells. Data were pooled from 10 videos imaged in four mice. (G) Top, schematic of experimental design. Bottom left, 72-μm z-projection view of TPLSM intravital imaging of mice imaged 2 h after GFP^+^ T cells transfer. Bottom right, mean velocities and straightness of migration path of the transferred cells. Data in (G) are representative of one of three independent experiments performed. Error bars represent SD. See also Video S7. (H) Similar analysis as in (A), performed 24 h after T cell transfer. Shows one of three representative experiments. Error bars represent SD. ^∗∗∗∗^p < 0.0001; ^∗∗∗^p < 0.001; ^∗∗^p < 0.01.

To revisit this point, we injected mice with blocking antibodies against LFA-1 and VLA-4 and tested lymphocyte entry using *in vivo* antibody labeling to mark cells exposed to blood and that therefore have not yet entered the white pulp (Cinamon et al., 2008). Blocking LFA-1 alone or in combination with VLA-4 reduced, but did not prevent, T and B cell entry into the white pulp (Figure 5A). Immunohistochemistry confirmed these results (Figure 5B). To better understand the mechanisms involved, we transferred GFP-expressing T cells and visualized their behavior 2 h after injecting integrin-blocking antibodies. Live imaging revealed no apparent effect of integrin blockade in terms of T cell association with the PT-tracks, nor on one-directional migration upon them (Figures 5C–5E; Video S7). However, migration velocities dropped by ∼50% compared with controls. A modest but consistent reduction in track straightness was also noted (Figure 5F). Similar behavior was observed when mice were injected with blocking antibodies 2 h prior to T cell transfer (Figure 5G; Video S7). These findings showed that, in the spleen, integrins promoted T cell homing by accelerating migration speeds, possibly by enhancing tangential traction forces exerted by migrating T cells on the surface (Hons et al., 2018). Based on these findings, we expected that the effect of integrin blockade would be time-dependent, delaying but not preventing entry. In agreement, the homing of T cells into the splenic white pulps of control and integrin-blocked hosts was similar at 24 h post-transfer (Figure 5H). These findings clarify the role of integrins in T cell entry to splenic T zones and help to explain why some studies have missed the contribution of these molecules in supporting this process (Nolte et al., 2002).

Video S7. Intravital Imaging of T Cell Migration along PT-Tracks in Mice Injected with LFA-1- and VLA-4-Blocking Antibodies, Related to Figure 5 Intravital imaging of GFP^+^ T cells transferred into hCD2-*DsRed* hosts prior to and after the injection of LFA-1- and VLA-4-blocking antibodies. In the first part of the video, cells were injected 2 h prior to the injection of LFA-1- and VLA-4-blocking antibodies. The spleens were imaged intravitally 2 h later. Many transferred T cells can be seen migrating along the PT-tracks toward, and accumulating within, a T zone. The trajectories of manually tracked transferred T cells are shown, with cells migrating toward the T zone highlighted in cyan and cells moving away from it in purple. All the transferred cells that were associated with PT-tracks and that could be tracked for at least 10 min are shown. In the second part of the video, GFP^+^ T cells were transferred 2 h after the injection of the blocking antibodies. As in the first example, GFP+ T cells associate and migrate along the PT-tracks. Elapsed time is shown as min:sec. Data are representative of at least six mice and eight videos.

### CCR7 Is Critical for One-Directional Migration and Entry into T Zones, but Is Not Essential for Attachment to PT-Tracks

**Figure 6:**
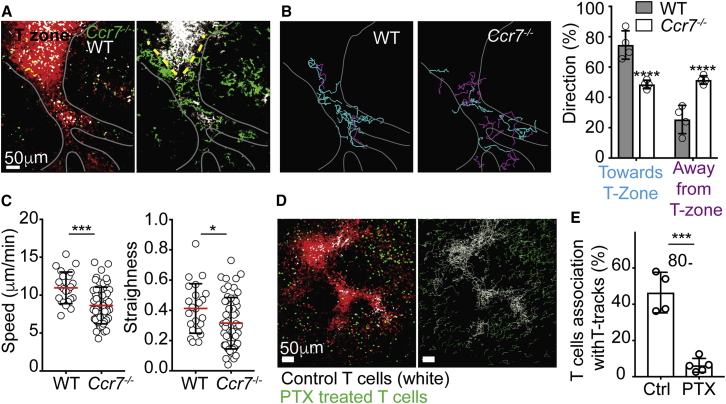
CCR7 Is Dispensable for T Cell Access to the PT-Tracks, but Required for Directional Migration and Entry into the T Zone (A) 84-μm z-projection (left) and time-projection (right) view showing migration of wild-type (WT, white) and *Ccr7*^−/−^ (green) T cells (green) 24 h after transfer into a hCD2*-DsRed* recipient. Grey line highlights PT-tracks connected to a T zone. Yellow dotted line indicates the “upper perimeter of the T zone” area, where *Ccr7*^−/−^ accumulate. See also Video S8. (B) Trajectories of transferred WT and *Ccr7*^*−/−*^ T cells moving toward the T zone (marked in cyan) and away from it (purple). Right, summary of frequencies of cells migrating toward or away from the T zones. Each circle represents the average frequency measured in one mouse. Error bars represent SD. (C) Mean velocities and straightness of cell trajectories of transferred T cells migrating along PT-tracks. The figure shows results from four independent experiments in which WT and *Ccr7*^*−/−*^ T cells were co-transferred. These data are consistent with those observed in four additional experiments where Ccr7^−/−^ T cells were transferred alone (data not shown). Error bars represent SD. (D) 105-μm z-projection view (left) and T cell tracks (right) showing migration of control PBS- (white) and pertussis toxin (PTX)-treated T cells (green) transferred into hCD2*-DsRed* recipient. (E) Frequencies of transferred cells associated with PT-tracks (right). Data are representative of five videos and three mice. Error bars represent SD. ^∗∗∗∗^p < 0.0001; ^∗∗∗^p < 0.001; ^∗^p < 0.05.

The chemokine receptor CCR7 is the main GPCR known to play a critical role in T cell entry to the spleen (Förster et al., 1999). However, the precise step it mediates during the entry process is not known. To address this, we visualized the behavior of *Ccr7*^*−/−*^ T cells. While *Ccr7*^*−/−*^ T cells were able to associate with PT-tracks and migrate upon them, they lost directionality and were moving toward and away from the T zone in similar frequencies (Figures 6A and 6B; Video S8). The velocities and straightness of *Ccr7*^*−/−*^ T cell trajectories were also reduced (Figure 6C). Yet, although approximately half of the *Ccr7*^*−/−*^ T cells reached the end of the PT-tracks, they were unable to progress beyond this point and complete entry (Figure 6A; Video S8). These results revealed an additional step in T cell entry to T zones, suggesting that detachment from the PT-tracks and migration deep into the T zone compartment is a regulated process that requires the chemokine receptor CCR7. It is possible that, at this location, additional chemotactic signals may be secreted, presenting opposing signals that retain cells locally in such a way that CCR7 is necessary to negate these signals and allow the cells to continue into the T zone. Additional studies are needed to test this hypothesis and determine whether this junction represents a functional checkpoint, where newly recruited T cells initially interact with local dendritic cells to scan antigenic material.

Video S8. Migration of CCR7 KO along PT-Tracks, Examples 1 and 2, Related to Figure 6 Two examples of time lapse series from intravital TPLSM showing migration of CCR7 KO T cells (green) 24 h after transfer into a hCD2dsRed recipient. Many CCR7 KO T cells are seen migrating along PT-tracks. However, the KO cells do not accumulate in the T zone and instead are seen hovering above the perimeter of the compartment. CCR7 KO T cells were found to migrate to and from the T zone with similar frequencies. CCR7 KO T cells associated with PT-tracks remained relatively straight, despite loss of directionality. In the first time sequence, three examples of T cell behaviors are highlighted by trajectories over time (purple lines, cells moving away from the T zone; and cyan line, cell moving toward). A summary of trajectories of migrating transferred T cells is displayed at the end of this sequence. Data are representative of at least four mice and eight videos. In the second example, a time lapse series of an 84-μm z-projection view show the migration of wild-type (white) and CCR7 KO (green) T cells (green) 24 h after co-transfer into a hCD2DsRed recipient. White line highlights PT-tracks connected to a T zone. Both WT and CCR7 KO cells are found migrating along the PT-tracks, but only WT T cells enter the T zone, while KO cells are unable to dive deep into the compartment and complete entry. In the end of this time sequence, a time projection is shown to highlight the cumulative trajectories of WT and CCR7KO T cells co-transferred. Data are representative of at least four mice and six videos. Elapsed time is shown as min:sec.

The above results demonstrated that CCR7 is critical for T cell migration into splenic T zones, but not for attachment to the PT-tracks, indicating additional factors must be involved. To obtain insight into possible mechanisms, we pre-treated T cells with pertussis toxin (PTX), which blocks signaling by GPCRs and thus strongly inhibits migration of lymphocytes (Spangrude et al., 1985). Purified GFP^+^ T cells were treated with PTX for 2 h and co-transferred with CMAC-labeled control (untreated) purified T cells into hCD2*-DsRed* hosts. While control T cells accumulated in PT-tracks and entered T zones, PTX-treated cells were unable to attach to these structures and were instead distributed in the red pulp (Figures 6D and 6E). Consequently, PTX-treated cells remained excluded from T zones. These results show that the localization of T cells to the PT-tracks is an active process and that additional GPCRs beyond just CCR7 are required for T cell entry into splenic T zones. A revised multi-step model of entry is proposed (Figure S5).

### The PT-Tracks Are Rapidly Modified during Inflammation, Restricting T Cell Entry into T Zones

Previous studies have shown that T cell entry into splenic white pulps is temporarily blocked during immunological challenges and that this correlates with reduction in the expression levels of the homeostatic chemokine CCL21 (Benedict et al., 2006, Mueller et al., 2007). However, there is currently no direct evidence that the reduced expression of CCR7 ligands is the principle mechanism that restricts the cell entry, and it remains possible that additional contributing factors may be involved. Furthermore, in view of new findings reported herein, reductions in CCR7 ligand availability alone might still allow T cells to reach the upper perimeter of the T zone compartment (as we observed with *Ccr7*^*−/−*^ T cells, Figure 6A), where functional interactions may take place. Alternatively, it may be that during immune responses, additional entry steps are blocked, thereby displacing cells to the red pulp where they are unlikely to be engaged in functional interactions. Thus, defining the precise step in which T cell entry fails during immunization may provide an important insight into the mechanisms and functional relevance of immune shutdown in the spleen.

**Figure 7:**
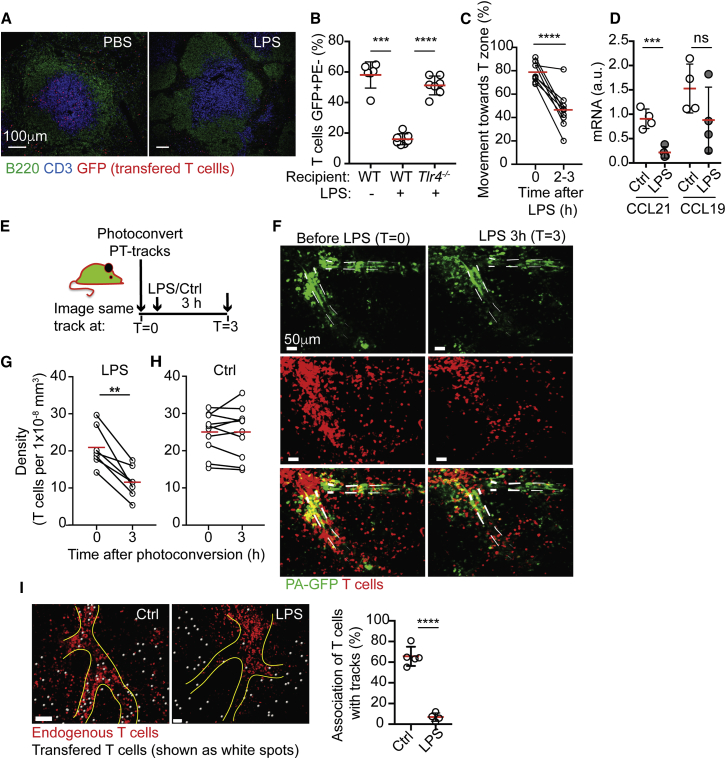
T Cell Entry to Splenic T Zones Is Impaired during Inflammation (A) Immunofluorescence analysis of spleens from control (PBS)- or lipopolysaccharide (LPS)-treated mice transferred with purified GFP^+^ T cells (red) and stained with anti-B220 (green) and anti-CD3 (blue). Mice were treated with LPSs or PBS for 6 h prior to transfer of purified labeled T cells. Analysis was performed 2.5 h post-cell-transfer. Data are representative of three independent experiments. (B) Flow-cytometric detection of GFP^+^ T cells labeled 5 min *in vivo* with anti-CD3-PE transferred into wild type (WT) or *Tlr4*^*−/−*^ recipients that were treated as in (A). Shown pooled data from two independent experiments. Error bars represent SD. (C) hCD2*-DsRed* mice were transferred with GFP^+^ T cells and imaged before (t = 0) and 2–3 h after LPS treatment. Individual PT-tracks were followed and the frequencies of cells migrating toward or away from the T zones on each track were determined. Each circle represents the average frequency measured in one PT-track before and after LPS administration. Figure shows results from 5 mice. (D) Quantitative PCR analysis of CCL21 and CCL19 in the stromal cell fraction of PBS- or LPS-treated mice. Data are representative of five independent experiments. Error bars represent SD. (E–G) hUbi-*PA-GFP*^+/−^ hCD2*-DsRed*^+/−^ mice were photoconverted to highlight individual PT-tracks. Images were collected before and 3 h after LPS administration. (E) Schematic of experimental design. (F) 60-μm z-projection view showing examples of two photoconverted PT-tracks monitored over time. Top, photoconverted cells shown in green. Grey lines highlight photoconverted PT-tracks. Middle, endogenous T cells within the same field of view, red. Bottom, overlap of photoconverted and endogenous T cells. (G) density of endogenous T cells along each PT-track. (H) Density of endogenous T cells in hUbi-*PA-GFP*^+/−^ hCD2*-DsRed*^+/−^ mice that were photoconverted and imaged as above without LPS treatment. Each circle in (G) and (H) represents the average frequency of cells measured in association with one PT-track. Figure shows results from three mice. (I) Left, snapshot from intravital imaging showing GFP^+^ T cells transferred into control (untreated) and LPS-treated hCD2*-DsRed* recipients. PT-tracks in LPS-treated mice were identified using PA-GFP photo-conversion, as above. T cells are highlighted using spots created with Imaris (white). Right, frequencies of transferred cells associated with PT-tracks. Each circle represents average frequency per mice. Error bars represent SD. Data are representative of at least seven videos and four mice per conditions. ^∗∗∗∗^p < 0.0001;^∗∗∗^p < 0.001; ^∗∗^p < 0.01.

In previous works, reduced T cell homing to splenic T zones was studied in the context of adaptive immune responses, with entry defects becoming evident several days post-infection or immunization (Benedict et al., 2006, Mueller et al., 2007). Since it is currently technically impossible to follow changes of cell behavior along the same PT-track within spleens of live mice over the course of multiple days, we first tested whether a similar splenic shut down is induced more rapidly in response to innate stimuli. To address this, we transferred GFP^+^ T cells into mice that had been injected with lipopolysaccharides (LPSs) 4–6 h earlier. Homing into T zones was determined 2.5 h post-transfer by histology and *in vivo* labeling. Under these conditions, frequencies of T cells that entered T zones were reduced (Figures 7A and 7B). Expression of Toll-like receptor 4 (TLR4) in host cells was necessary for this effect, indicating the importance of environmental components in restricting entry (Figure 7B). In line with this possibility, homing of T cells that have been exposed to LPSs *in vivo* prior to transfer into control recipients showed only modest reduction in entry, further pointing to external modification of extrinsic factors in limiting entry (Figure S6A). To directly determine which step is affected, we transferred purified GFP^+^ T cells into hCD2*-DsRed* hosts and monitored cell behavior along the same PT-track before and after LPS injection. We found that within 2–3 h post-LPS treatment, T cells that were associated with PT-tracks were moving in random directions to and from T zones, in a manner similar to *Ccr7*^−/−^ T cells (Figure 7C). This observation was consistent with reduced CCL21 mRNA levels in the splenic stromal cell fraction (Figure 7D), indicating that loss of CCR7 ligands likely contributed to reduced entry. However, during these experiments, we also noted that by 3–4 h post-LPS treatment, our ability to find clear paths of endogenous T cells within inflamed spleens declined. This suggested the possibility that, in addition to reduction in expression of CCR7 ligands, inflammation may also limit recruitment of newly arriving T cells to the PT-tracks, reducing the frequency of visible structures. To more directly test this hypothesis, we next optimized an approach that would allow us to stably label the PT-tracks prior to LPS treatment in live animals by photoconverting regions in mice ubiquitously expressing a photoconvertible GFP (PA-GFP) (Victora et al., 2010). hUbi-*PA-GFP* hCD2*-DsRed* double transgenic mice were intravitally imaged to identify clear PT-tracks that were subsequently photoconverted (Figure 7E). While in these mice, photo-conversion also labeled T cells that were located within the target area, over time these cells migrated away from the field of view, leaving the stable structural elements highlighted to serve as landmarks (Figures 7F and S6B). This approach allowed us to quantitatively determine changes in endogenous T cell density associated with individual PT-tracks over time. We found that LPSs induced rapid reduction in the concentration of T cells associated with PT-tracks, with an average loss of approximately 50% of the cells within 3 h post-treatment (Figure 7G). In contrast, the density of endogenous T cells associated with photoconverted PT-tracks in control mice that did not receive LPSs remained stable over similar periods of time, confirming the dependence upon this innate cue (Figure 7H). In line with these observations, live imaging of newly transferred T cells into LPS-treated mice further confirmed defects in association with PT-tracks, leading to displacement of cells to the red pulp (Figure 7I), similar to the distribution observed with PTX-treated cells (Figure 6D). Together, these results showed that in addition to loss of chemotactic gradients along the PT-tracks, entry arrest was mediated via changes that occurred at the initial step of attachment to them, thus indicating the involvement of an additional factor that is needed for this step. These observations further raise the possibility that under certain conditions, migration within the lymphoid tissue may be regulated independently from entry events, such that the highly organized nature of the compartment, which is necessary for adaptive immune responses, can remain intact while entry of new T cells is restricted. Further studies exploring the chemotactic cues expressed by the PT-track-associated stromal cells under homeostatic and inflammatory conditions will help to address this hypothesis and define the precise mechanisms involved.

## Discussion

The BCs were first discovered over four decades ago as “channel like” regions, where lymphocyte movement in and out of white pulps is hypothesized to occur (Mitchell, 1973). Whether these regions allow cell passage simply due to the absence of a barrier or whether they contain elements that support directional migration remain unresolved. Furthermore, since no clear paths have been described in the red pulp in subsequent studies, the manner by which cells find their way to these “open channels” is unknown. The lack of clarity in understanding the structural composition of the BCs and how cells migrate through them introduces confusion in the field, and for the past 45 years, models have described the BCs as “breaks in the MZ shell,” hypothesizing that cells “drift” toward these sites with the blood flow and are recruited into the T zone by contacting TRCs that extend through them. It has also been hypothesized that the BCs act as a point of both entry and egress, giving rise to the notion that splenocytes enter and leave the white pulp via the same route. Here we used advanced imaging approaches to visualize T cells circulating in the spleens of live mice in order to directly explore this process. We found that while the BCs served as ports of entry into the white pulp, the actual entry paths that supported migration into T zones were guided by blood vessels that acted as scaffolds for a network of stroma-coated routes. We propose to refer to these paths as “perivascular T-tracks” (PT-tracks) to reflect their functional and structural characteristics. In contrast to the current hypothesis, T cells were not “flushed” toward the BCs passively by the blood flow, but instead were first guided to them in a process that was independent of CCR7 but required activation of GPCRs. Once attached to the PT-tracks, CCR7 promoted one-directional movement of T cells toward the T zone followed by detachment from them and migration into the T zone. This final step of entry was also regulated by CCR7, possibly reflecting the existence of a checkpoint located at the gate of the compartment. Integrins played a role in enhancing migration speeds during this process, but were not required for attachment or adhesion to the PT-tracks. Using intravital live imaging was crucial for our dynamic and structural analysis because we found that the vascular structures that form the PT-tracks collapsed during *ex vivo* processing of splenic tissue sections. This challenge likely explains why previous studies using static section analysis were limited in their ability to explore the entry routes within this organ.

Our study established that movement along PT-tracks supports entry into T zones, but not egress from them. However, despite extensive efforts, we were not able to identify the exit routes. We hypothesize that this process may take place in deeper regions of the spleen beyond our imaging range. Alternatively, egress may occur via structures that overlap with the T zone compartment, such that the high density of cells masks visualization of egress events. Better definition of factors that regulate entry will allow temporal control of cell trafficking in the spleen and may provide an approach to define the path T cells take to leave splenic T zones.

The finding that CCR7, LFA-1, and VLA-4 were not required for attachment to the PT-tracks suggests that additional chemotactic or adhesion molecules promote this process. While the adhesion receptor Clever-1, which was shown to enhance homing of lymphocytes into splenic white pulps (Tadayon et al., 2019), may contribute to the process, other factors are likely involved, as Clever-1^−/−^ mice showed only partial defects in T cell entry. In agreement, we found that PTX-treated T cells failed to associate with the PT-tracks and instead distributed in the red pulp. These observations are in line with previous work showing a similar effect of PTX treatment on T cell distribution in spleen sections (Cyster and Goodnow, 1995). Given that the major homeostatic GPCRs, including CXCR5 and CXCR4, are not essential for entry or attachment to PT-tracks, it may be that this step requires another, yet unidentified, GPCR. Alternatively, attachment to the PT-tracks may depend on the cumulative effect of several receptors, as was demonstrated in the case of entry to LNs and peripheral sites (Calderón and Boehm, 2011, Dupré et al., 2015, Okada et al., 2002). Detailed analysis of the unique reticular cell subsets that form the base of the PT-tracks will help to further address this question and discover new factors that promote T cell entry under steady-state conditions and during immune responses.

In contrast to LNs, where CCR7 is needed to trigger integrin-mediated firm adhesion and attachment to HEVs during entry, in the spleen, the same molecules were necessary to promote directional migration along the PT-tracks, but not for attachment to them. This difference likely reflects the unique challenges T cells face during entry to these organs; while migration into LNs depends on the ability of the cells to slow down before transmigrating directly into the parenchyma of the tissue, in the spleen, the cells are initially released from the blood passively, and their main challenge is to navigate from these locations to the white pulp passing via blood-exposed regions that restrict cellular migration. We hypothesize that the multi-layered organization of the reticular cell network coating the PT-tracks may provide a partially sheltered environment, where exposure to high rates of blood flow is limited. In support of this possibility, we found that PT-track-associated cells were not fully accessible to bloodstream-restricted antibodies and that blocking integrins did not displace cells from them. Thus, T cells use similar molecules to enter LNs and spleen, but the role these factors play during entry, and the structural elements the cells use, differ.

While under steady-state conditions the PT-tracks exclusively mediated entry, it remains possible that during immunological challenges these structures can be modified to provide exit routes for activated T cells. In support of this hypothesis, effector CD8^+^ T cells have been shown to increase expression of CXCR3 and accumulate within BC-like structures in the spleens of infected mice (Hu et al., 2011, Khanna et al., 2007, Kurachi et al., 2011, Shah et al., 2015). During these responses, extrinsic changes in the splenic environment (Benedict et al., 2006), including reduced expression of the homeostatic chemokine CC21a (Benedict et al., 2006, Mueller et al., 2007), were shown to temporarily block entry of naive T cells to T zones (Benedict et al., 2006, Mueller et al., 2007). Here, we found that a similar entry blockade and reduction in CCL21 expression occurred during acute inflammation induced by LPSs. Under these conditions, one-directional migration of circulating T cells along the PT-tracks was impaired, indicating a change in chemotactic gradients within these paths. Furthermore, recruitment of circulating T cells to the PT-track was reduced, likely reflecting the loss of additional GPCR ligands necessary for this step. It is therefore tempting to speculate that during certain conditions, changes in chemotactic gradients along the PT-tracks may lead to a temporal shift in the directionality of T cell migration along them, denying newly arriving cells from entering T zones while supporting egress of activated cells to the red pulp. Developing mouse models that will allow direct visualization of the PT-tracks for prolonged periods of time will help to directly test this hypothesis.

Finally, our study revealed a function for the splenic vasculature in supporting intra-organ trafficking of circulating T cells. A similar mode of “vascular-guided” (also known as “vasophilic”) migration has been shown to play a key role in neuroblast navigation during development and regeneration (Bovetti et al., 2007, Saghatelyan, 2009, Segarra et al., 2015) and was also suggested to mediate lymphocyte movement in tumor sites, where cytotoxic T lymphocytes were found to migrate following collagen fibers and blood vessels (Boissonnas et al., 2007). Our study revealed that this atypical mode of migration is extensively used by lymphocytes, not only in the context of pathological conditions, but also during immune homeostasis. It remains to be determined whether vasophilic migration directs lymphocyte migration in other tissues or whether human spleens, where analogous structural constrains exist, evolved similar mechanisms to support cell trafficking. Using live imaging and improved approaches to conserve fragile structural elements in static sections may help to address these questions.

## STAR★Methods

### Key Resources Table

**Table:** 

REAGENT or RESOURCE	SOURCE	IDENTIFIER
**Antibodies**		

Biotin B220, clone RA3-6B2	Biolegend	Cat #: 103204; RRID: AB_312989
Biotin Cd11b, clone M1/70	Biolegend	Cat #: 101204 RRID: AB_312787
Biotin Ly6G, clone 1A8	Biolegend	Cat #: 127604; RRID: AB_1186108
Biotin CD49b, clone DX5	Biolegend	Cat #: 108904; RRID: AB_313411
PE CD45, clone 30F11	Biolegend	Cat #: 103106; RRID: AB_312971
PerCP/Cy5.5 TER119	Biolegend	Cat #: 116227; RRID: AB_893638
PE CD19, clone 6D5	Biolegend	Cat #: 115508; RRID: AB_313643
PE CD3, clone 17A2	Biolegend	Cat #: 100206; RRID: AB_312663
Alexa700 B220, clone RA3-682	Biolegend	Cat #: 103232; RRID: AB_493717
APC CD3, clone 145-2C11	Biolegend	Cat #: 100236; RRID: AB_2561456
BV785 CD4, clone RM4-5	Biolegend	Cat #: 100552; RRID: AB_2563053
BV605 CD8, clone 53-6.7	Biolegend	Cat #: 100744; RRID: AB_2562609
APC-Cy7 CD45, clone 30-F11	Biolegend	Cat #: 103116; RRID: AB_312981
APC-Cy7 TER119, clone TER-119	Biolegend	Cat #: 116223; RRID: AB_2137788
APC CD31, clone MEC13.3	Biolegend	Cat #: 102410; RRID: AB_312905
PE Podoplanin, GP38, clone 8.1.1	Biolegend	Cat #: 127408; RRID: AB_2161928
Integrin alpha4, CD49d, clone PS/2	BioXcell	Cat #: BE0071; RRID: AB_1107657
Integrin alphaL, CD11a, clone M17/4	BioXcell	Cat #: BE0006; RRID: AB_1107578
FITC CD169, clone 3D6.112	BIO-RAD	Cat #: MCA884FT; RRID: AB_1100895
GFP, rabbit polyclonal	Thermo Fisher	Cat #: A-11122; RRID: AB_221569
Alkaline Phosphatase anti-Fluorescein, polyclonal	Sigma	Cat #: 11426338910 Roche; RRID: AB_2734723
HRP anti-rabbit, polyclonal	Thermo Fisher	Cat #: 31460; RRID: AB_228341
Alexa647 CD169, clone 3D6.112	BIO-RAD	Cat #: MCA947A647; RRID: AB_10545834
CD31, clone MEC13.3	BD Biosciences	Cat #: 557355; RRID: AB_396660
Biotin CD140b, PDGFR beta, clone APB5	Biolegend	Cat #: 136010; RRID: AB_2236916
ER-TR7, clone ER-TR7	BioXcell	Cat #: BE0253; RRID: AB_2687734
Biotin CCL21, polyclonal	R&D systems	Cat #: BAF457; RRID: AB_2072082
Podoplanin, GP38, clone 8-1-1	eBioscience	Cat #: 14-5381-82; RRID: AB_1210505
Alexa488 anti-rat IgG	Thermo Fisher	Cat #: A21208; RRID: AB_2535794
eFluor660 anti-rat IgG	Thermo Fisher	Cat #: 50-4017-82; RRID: AB_2574211
Alexa488 anti-rabbit igG	Thermo Fisher	Cat #: A-11008; RRID: AB_143165
Alexa647 anti-Armenian hamster IgG	Jackson ImmunoResearch	Cat #: 127-605-160; RRID: AB-2339001
Biotin CD4	Biolegend	Cat #: 100508; RRID: AB-312711
Alexa647 B220, clone RA3-6B2	Biolegend	Cat #: 103226; RRID: AB-389330
Cy3 aplphaSMA, clone 1A4	Sigma	Cat #: C6198-100UL; RRID: AB-476856
Rabbit anti-EYFP living colors full length GFP, polyclonal	Takara	Cat #: 632592; RRID: AB-2336883
Goat anti-CCL21	R&D systems	Cat #: AF457; RRID: AB-2072083
Rat anti-ER-TR7	Abcam	Cat #: ab51824; RRID: AB-881651
Alexa488 anti-rabbit IgG	Jackson ImmunoResearch	Cat #: 111-547-003; RRID: AB-2338058
Dylight549 anti-rat IgG	Jackson ImmunoResearch	Cat #: 112-505-175; RRID: AB-2338335
Dylight549 anti-Syrian Hamster IgG	Jackson ImmunoResearch	Cat #: 307-506-003; RRID: AB-2339595

**Critical Commercial Assays**		

Alexa488 streptavidin	Invitrogen	Cat #: S11223
Cy3 streptavidin	Jackson ImmunoResearch	Cat #: 016-160-084
Alexa647 streptavidin	Jackson ImmunoResearch	Cat #: 016-600-084
BioMag Goat anti-rat IgG	QIAGEN	Cat #: 310107
CFSE 5-(and 6-)-carboxyfluorescein diacetate succinimidyl ester	Life Technologies	Cat #: C1157
Cell tracker blue probe CMAC 7amino4chloromethylcoumarin	Life Technologies	Cat #: C2110
Cell Tracker orange CMTMR	Life Technologies	Cat #: C2927
Pertussis Toxin Islet Activating Protein Salt-Free (PTX)	Quadratech Diagnostics Ltd.	Cat #: 181
LPS, *Escherichia coli* 0111:B4	Sigma Aldrich	Cat #: L4391
Dextran-FITC 2000KDa	Sigma Aldrich	Cat #: FD2000S
Collagenase P	Sigma Aldrich	Cat #: 11213857001
Dispase I	Sigma Aldrich	Cat #: D4818-2MG
MACS TER119 microbeads	Miltenyi Biotec	Cat #: 130-049-901
MACS CD45 microbeads	Miltenyi Biotec	Cat #: 130-052-301

**Experimental Models: Organisms/Strains**		

Actin-*CFP* mice, Tg(ACTB-*ECFP*)1Nagy/J	Jackson Laboratory	004218
*Ccr7*^*−/−*^ mice, B6.129P2(C)-*Ccr7*tm1Rfor/J	Jackson Laboratory	006621
Ubi-*GFP* mice, C57BL/6-Tg(UB C-*GFP*)30Scha/J	Jackson Laboratory	004353
Rosa26-*YFP* mice, B6.129X1-Gt(ROSA)26Sortm1(EYFP)cos/J	Jackson Laboratory	001648
CD19^*cre/cre*^ mice, B6.129P2(C)-Cd19tm1(*Cre*)Cgn/J	Jackson Laboratory	006785
*Tlr4*^*−/−*^ mice, B6(Cg)-*Tlr4*^*tm1.2Karp*^/J	Jackson Laboratory	029015
Ubi-*PA-GFP* mice, B6.Cg-Ptprc^a^Tg(UBC-*PA-GFP*)1Mnz/J	Jackson Laboratory	022486
hCD2-*DsRred* mice	Veiga-Fernandes et al., 2007	N/A
VE-cadherin-*tdTomato*, C57BL/6-Tg(Cdh5-*cre/ERT2*)rosa26-*TdTomato*	Kindly provided by Dr. Bostjan MArkelc, Oxford University	N/A
BAC-transgenic C57BL/6-Tg(Ccl19-*tTA*)^687BIAT^	Available under MTA, laboratory of Dr. Burkhard Ludewig	N/A
LC1	Schönig et al., 2002	N/A

**Oligonucleotides**		

qPCR primers Ccl19 FP: CTG CAA GAG AAC TGA ACA GAC	Life Technologies	N/A
qPCR primers Ccl19 RP: CTT CTG ACT CTC TAG GTC TAC	Life Technologies	N/A
qPCR primers Ccl21 FP: ATCGTGAAAGCCTTCGCTACCTT	Life Technologies	N/A
qPCR primers Ccl21 RP: GCTGTTGCCTTTGTTCTTGGCAGA	Life Technologies	N/A
qPCR primers GAPDH FP: AGGTCGGTGTGAACGGATTTG	Life Technologies	N/A
qPCR primers GAPDH RP: TGTAGACCATGTAGTTGAGGTCA	Life Technologies	N/A
QuantiTect Primers Hprt	QIAGEN	QT00166768
QuantiTect Primers Ccl19	QIAGEN	QT02532173
QuantiTect Primers Ccl21a	QIAGEN	QT00284753

**Software and Algorithms**		

FlowJo 9.7.1 (Treestar inc)	https://www.flowjo.com/	RRID: SCR_008520
Graphpad prism version 8	https://www.graphpad.com/scientific-software/prism/	RRID: SCR_002798
Adobe illustrator CS6	http://www.adobe.com/products/illustrator.html	RRID: SCR_010279
Imaris 9.2.1	http://www.bitplane.com/imaris/imaris	RRID: SCR_007370
Fiji, ImageJ, NIH Bethesda	http://fiji.sc/	RRID: SCR_002285
ZEN Digital Imaging for Light Microscopy	http://www.zeiss.com/microscopy/en_us/products/microscope-software/zen.html#introduction	RRID: SCR_013672

### Resource Availability

#### Lead Contact

Further information and requests for resources and reagents should be directed to and will be fulfilled by the lead Contact, Tal Arnon (tal.arnon@kennedy.ox.ac.uk).

#### Materials Availability

*Ccl19*-tTA.2 mouse strain is available under an MTA from the authors.

#### Data and Code Availability

This study did not generate/analyze [datasets/code].

### Experimental Model and Subject Details

#### Mice

Male and female mice aged 6-24 weeks were used for all experiments. Mice were age-matched and sex-matched within each individual experiments. Wild-type C57BL/6 mice were purchased from Charles River. β-actin*-CFP* (004218; Tg(ACTB-*ECFP*)1Nagy/J), *Ccr7*^*−/−*^ (006621; B6.129P2(C)-*Ccr7*tm1Rfor/J), hUbi-*GFP* (004353; C57BL/6-Tg(UB C-*GFP*)30Scha/J mice), Rosa26-*eYFP* (001648; B6.129X1-Gt(Rosa)26Sortm1(*EYFP*)cos/J), CD19^cre/cre^ (006785; B6.129P2(C)-CD19*tm1(Cre)*Cgn/J), *Tlr4*^*−/−*^ (029015;B6(Cg)-*Tlr4*^*tm1.2Karp*^/J), hUbi-*PA-GFP* (022486; B6.Cg-Ptprc^a^Tg(UBC-*PA-GFP*)1Mnz/J) were purchased from The Jackson Laboratory and backcrossed for at least 10 generations to a C57BL/6 strain. To generate CD19^cre/cre^ Rosa*26*^*YFP/YFP*^, CD19^*cre/cre*^ mice were crossed to Rosa26-*eYFP* animals. To generate hUbi-*PA-GFP* hCD2-*DsRed*, hUbi*-PA-GFP* mice were crossed to hCD2-*DsRed* animal. C57BL/6 hCD2-*DsRred* (Veiga-Fernandes et al., 2007) were a kind gift form Professor Fiona Powrie’s lab at Oxford Univeristy. VE-cadherin-*tdTomato* mice (C57BL/6-Tg(Cdh5*-cre/ERT2*)Rosa26-*TdTomato*) were kindly provided by Dr. Bostjan Markelc from Oxford University. Mice were treated with tamoxifen to induce tdTomato expression (Sörensen et al., 2009). The BAC-transgenic C57BL/6N-Tg(Ccl19*-tTA*)^687 BIAT^ (Ccl19*-tTA*.v2) mouse was generated using the homologous recombination-mediated transgenesis method (Sparwasser and Eberl, 2007) and further crossed with B6.129X1-Gt(Rosa)26*Sor*^*tm1(EYFP)Cos*^/J (R26R-*EYFP*) mice from the Jackson Laboratories and the LC1 strain (Schönig et al., 2002) (to generate the Ccl19-*iEYFP.2* mouse model. Both Ccl19-*iEYFP* and Ccl19-*iEYFP.2* mouse models were generated with the same BAC clone with lower transgene penetrance and more restricted transgene expression in the Ccl19-*iEYFP.2* strain. While the Ccl19-*iEYFP* mouse model highlights all white pulp fibroblasts (Cheng et al., 2019) the Ccl19-*iEYFP.2* mouse model predominantly labeled the fibroblastic reticular cells in the T cell zone (Supplementary fig. S4).

Animals were bred and maintained under specific pathogen-free (SPF) conditions in accredited animal facilities at Kennedy Institute of Rheumatology, University of Oxford and experiments were in accordance with the UK Scientific Procedures Act (1986) under a Project License (PPL) authorized by the UK Home Office.

### Method Details

#### Chimera generation

For generation of chimeras, 8 weeks old B6 mice were irradiated twice with sublethal dose (450rads) 4 h apart followed by intravenous injection of at least 5 million bone marrow cells per mouse.

#### Cell isolation and adoptive transfers

T cells were enriched by negative selection. Cell suspension was labeled using a combination of anti-B220 (RA3-6B2, Biolegend), anti-CD11b (M1/70, Biolegend), anti-Ly6G (1A8, Biolegend), and anti-DX5 (CD49b, Biolegend) antibodies followed by magnetic isolation using anti-rat BioMag beads (QIAGEN). B cells were enriched with a MagniSort mouse B cell enrichment kit (Affymetrix eBioscience). T and B cells were labeled with 10 μM of either CFSE, CMTMR for 10 min or 100uM CMAC (Invitrogen, life Technologies) for 30 min and 10 million of each were adoptively transferred intravenously. Unless otherwise specified, mice were imaged 24 h after transfer.

For the inhibition of Gi protein-coupled receptors assay, 20 millions GFP^+^ or CMAC labeled T cells were incubated 2 h at 37°C with 100ng per ml pertussis toxin (Quadratech Dagnostics Ltd.) or PBS, washed and 10 millions of each were co-injected into the tail vein of hCD2-*DsRed* mice few h before imaging.

To image the blood flow, blood from hUbi-*GFP* mice was collected in Alsever’s solution and washed with cold-PBS followed by a density gradient purification (Ficoll-Paque Plus). Cells at the bottom of the tubes were collected and the purity was assessed by flow cytometry based on staining with anti-CD45-PE (clone 30F11, Biolegend) and anti-TER119-PerCP-Cy5.5 (Biolegend). The purity was typically over 96%. Approximately 10^8^ red blood cells were injected intravenously minutes before imaging.

To highlight the vasculature, 100 μg Dextran Fluorescein 2000 kDa (Invitrogen) was injected intravenously immediately before acquiring time lapse of pre-identified regions of interest.

#### Preparation of splenic stromal cells

Spleens were harvested and perfused with RPMI 1640 medium containing 2% FCS, 20 mM HEPES (all from Lonza), 0.2 mg per ml Collagenase P (Sigma Aldrich), 0.8 U per ml Dispase I (Sigma Aldrich) and 100 μg per ml DNaseI (Applichem) with 22G syringe. Samples were torn into smaller pieces and incubated at 37°C for 30 min, with resuspension and collection of supernatant every 15 min to PBS containing 1% FCS and 10 mM EDTA (MACS buffer). To enrich fibroblastic stromal cells, hematopoietic and erythrocytes were depleted by incubating the cell suspension with MACS anti-CD45 and anti-TER119 microbeads (Miltenyi Biotec) and passing them through a MACS LS column (Miltenyi Biotec). Unbound single cell suspensions were used for further flow cytometric analysis.

#### Flow cytometry, *in vivo* labeling

*In vivo* labeling has been previously described (Cinamon et al., 2008). Briefly, mice were injected intravenously with 200 μL containing 2.5 μg of anti-CD19-PE (clone 6D5, Biolegend) and or anti-CD3-PE (clone 17A2, Biolegend) antibodies 5 min before culling the mice. Spleens were collected and stained on ice with anti-B220-Alexa700 (clone RA3-682, Biolegend), anti-CD3-APC (clone 145-2C11, Biolegend), anti-CD4-BV785 (clone RM4-5, Biolegend), anti-CD8-BV-605 (clone53-6.7, Biolegend). For stromal cells characterization of the Ccl19-*iEYFP.2* mice, cells were stained with antibodies against CD45 (clone:30-F11, BioLegend), Ter119 (clone:TER-119, BioLegend), CD31 (clone:MEC13.3, BioLegend), PDPN (clone:8.1.1, BioLegend).Cells were acquired on a BD LSR Fortessa (BD Biosciences) and analyzed on FlowJo software (Tree Star Inc.).

#### T cell homing in integrin blocked and LPS treated hosts

To block integrins, we injected 100 μg per mouse anti-α4 (clone PS/2, BioXcell) and anti-αL (clone M17/4, Bioxcell), intravenously. To induce acute inflammation, mice were injected intraperitoneally with 50 μg LPS (E. Coli 0111:B4, Sigma). Cells were injected into treated or untreated hosts and 2.5 h later, mice were *in vivo* labeled followed by analysis by flow cytometry and histology. In TPLSM experiments, the antibodies were injected either 2 h before or 2 h after cell transfer, as specified in the figure legends.

#### Immunohistochemistry

For immunohistochemistry, spleens were fixed with 4% PFA (Pierce 16% Formaldehyde (w/v), Thermo Scientific) in PBS for 2 h at 4 degrees, washed with PBS, incubated overnight with 30% sucrose in PBS, embedded in OCT (Agar Scientific, AGR1180) and snap frozen in dry ice with 100% methanol. 7 μm sections were cryostat-cut and collected on SuperFrost Plus Adhesion slides. Sections were allowed to dry for at least 1 h. Before staining, slides were rehydrated for 10 min in TBS (immunohistochemistry, IHC) and blocked with normal mouse serum (2%) for 20 min in a humidified chamber. Sections were stained with anti-CD169-FITC (clone 3D6, BIO-RAD) and anti-GFP followed by anti-FITC coupled to alkaline phosphatase (Sigma) and anti-rabbit-HRP (Jackson ImmunoResearch).

#### Intravital imaging of T cells in the spleen

All imaging experiments were done intravitally, in live mice, using two-photon laser-scanning microscopy (TPLSM) as previously described (Arnon et al., 2013). Briefly, Mice were anaesthetized using isoflurane (100% (v/v) inhalation vapor liquid, Zoetis). Flow rates of 4% and 1.5% were used to induce and maintain the mice under deep anesthesia, respectively. The bottom half of the left flank of the mouse was shaved, and a small skin incision was made below the costal margin above the spleen. A ∼1 cm cut was then made in the peritoneal cavity and the spleen was gently mobilized on its stalk. To stabilize the spleen during imaging, a spring-loaded platform (McDole et al., 2012) was placed over the mouse and screwed down until the cover glass made contact with the spleen capsule. The mouse was placed on a Biotherm stage warmer at 37 C (biogenics) and image using an upright Zeiss LSM880 microscope equipped with a W Plan-Apochromat 20x1.o DIC D = 0.17 (uv) vis-IR M27 75mm objective and two Mai Tai lasers with a range of excitation of 690-849 and 850-990nm. Detectors used include a nose piece GaAsp1 with filters allowing detection of 425/60nm for blue dyes and CFP, a nose piece GaAsp2 with 500-550nm filters for detection of green dyes including GFP, YFP and CFSE, and a BIG2 GaAsp detector with filters range of 590-610nm for red dyes and red fluorescent proteins. Videos were acquired with Zen software (Carl Zeiss, Inc.) Cell labeled with CMAC were exited with 810nm, whereas all other dyes and fluorescent proteins were excited with 930nm. Series of planes of 3um z-spacing spanning a depth of 50-110um were recorded every 15-30 s. Videos were made and analyzed using Imaris 9.2.1 (Bitplane) and ImageJ.

For photoconversion experiments, spleens of live hUbi-*PA-GFP*^+^ hCD2-*DsRe*d^+^ double transgenic mice were surgically exposed, as above, and PT-tracks were located by scanning at 930nm. Regions of inserts were irradiated using Mai Tai DeepSee laser at a wavelength of 840nm. Power intensity of the laser was between 30%–50% (equivalent to approximately 775-1300mW) using pixel dwell time of 132 μsec with 2-4 iterations, depending on the depth of the region of interest. Successful conversion was assessed at 930nm and the integrity of the converted region was evaluated based on motility of cells within it. Movies were acquired immediately following photoconversion (time 0). Mice were removed from the microscope and injected intravenously with either control (saline) or 50 μg LPS. Photoconverted regions were imaged again, at the indicated time points.

#### Immunofluorescence and vibratome sections

*Ex vivo* immunofluorescent analysis of PT-tracks was performed using thick (150-350 μm) vibratome sections of freshly fixed (unfrozen) spleens. Mice were perfused with fixative solution (PBS, 1%PFA, 0.07M L-lysine, 2.1mg per ml m-periodate, pH:7.4) (Sigma) directly through intracardial injection. Perfused spleens were collected and incubated for 24 h in the above fixative solution at 4 degrees. The full spleens were embedded in 2% agarose and longitudinal sections of 100-350um were cut using a vibratome (Ci Campden Instruments 5100 mz) at a frequency of 50Hz, an amplitude of 1.50mm and speed of 0.15mm per second. Sections were incubated with 0.5% Triton X-100 (Sigma) for 10 min followed by incubation with blocking solution containing PBS, 2%BSA, 2% mouse serum and 0.3% Triton X-100 for 2 h at room temperature. For staining, antibodies were diluted to a concentration of 5 μg per ml in the above blocking solution and allowed to incubate with the sliced sections for at least 24 h at 4 degrees. For staining of vibratome sliced sections we used anti-CD169-A647 (clone 3D6, AbD serotec), anti-CD31 (clone MEC13.3, Bioscience), anti-PDGFRβ-biotin (clone APB5, Biolegend), anti-ER-TR7 (clone ER-TR7, BioXcell), anti-GFP (ThermoFisher Scientific), anti-CCL21 (Goat polyclonal IgG, R&D systems) and anti-Podoplanin (clone 8-1-1, eBioscience) followed by secondary antibody anti-rat A488 (ThermoFisher Scientific), anti-rat eF660 (ThermoFisher Scientific), anti-rabbit A488 (BD Bioscience), anti-hamster A647 (Jackson ImmunoResearch), streptavidin-A488 (Invitrogen) Between primary and secondary stain, sections were washed three times for 2 h. Stained sections were mounted on slides using Fluoromount-G (Cambridge Bioscience) and imaged with a LSM880 Zeiss microscope.

Standard immunofluorescent histology (for characterization of Ccl19-*iEYFP.2* mouse line and analysis of spleen sections from LPS treated mice), spleens were fixed, frozen and sliced (Cheng et al., 2019). Sections were stained with anti-CD4 (BioLegend), anti-B220 (BioLegend), anti-CD169-A647 (clone 3D6, AbD serotec), anti-αSMA (Sigma), anti-EYFP (Takara), anti-CCL21 (R&D Systems) and anti-ERTR7 (abcam) followed by Alexa488-conjugated anti-rabbit-IgG, Dylight549-conjugated anti-rat-IgG, Dylight549-conjugated anti-syrian hamster-IgG, Cy3-conjugated Streptavidin, Alexa647-conjugated Streptavidin, Alexa647-conjugated anti-goat-IgG (all purchased from Jackson Immunotools).

#### RNA isolation and Real-time PCR

RNA was isolated using the RNA MiniPrep Kit (QIAGEN). To generate cDNA for qRT-PCR analysis, the High-Capacity cDNA Reverse Transcription Kit was employed (Applied Biosystems). Quantitative RT-PCR was performed using the Fast blue qPCR Master mix (Primerdesign Ltd.) on a Viia7 real time PCR system (Applied Biosystems)) using the following primers: *Ccl19*: CTG CAA GAG AAC TGA ACA GAC/ CTT CTG ACT CTC TAG GTC TAC, CCL21- ATCGTGAAAGCCTTCGCTACCTT/ GCTGTTGCCTTTGTTCTTGGCAGA, GAPDH:AGGTCGGTGTGAACGGATTTG/TGTAGACCATGTAGTTGAGGTCA for the experiments on inflammation and the QuantiTect Primers Hprt: QT00166768, *Ccl19*: QT02532173, Ccl21a: QT00284753 (QIAGEN, Venlo, Netherlands) were used for the characterization of cells sorted from the newly generated Ccl19-*iEYFP.2* mouse. Relative expression of samples was calculated by the comparative cycling threshold method (ΔΔ-CT method), using the expression of *gapdh* or *Hprt* for normalization.

#### Image acquisition and analysis

During imaging sessions we primarily focused on areas of interest, which typically included regions where a T zone could be clearly identified and where the location of the red pulp could be estimated with good approximation. In most experiments, we used the hCD2-*DsRed* mice, in which endogenous T cells express DsRed, to highlight the T zone and to delineate PT-tracks. The location of the red pulp was estimated based on cell behavior (i.e., being more rounded and immotile), proximity to the capsule and autofluorescence, which is enhanced in the resident macrophages-rich red pulp. In some cases, to identify the MZ, we delineated the follicular compartment based on high concentration of GFP^+^ (transferred) or YFP^+^ (endogenous) B cells. The MZ was defined as the area that immediately interfaces the FO, identified based on high density of follicular B cells migrating within them (Arnon et al., 2013). To extract migratory parameters of individual T cell behavior, we tracked GFP^+^ T cells transferred into hCD2-*DsRed* mice such that the endogenous T cells provided a landmark for both the T zone and the PT-tracks. Cells were tracked using Imaris, by creating seed points. All tracks were manually examined and verified. Migratory parameters were calculated using Imaris (Bitplane), MATLAB (MathWorks), or MetaMorph, as described before (Allen et al., 2007). To calculate circularity, cells were masked using Fiji (ImageJ, NIH, Bethesda) based on fluorescence intensity after thresholding the image. Area and perimeter were measured, and circularity was calculated on the basis of 4pi (area/perimeterˆ2).

In some cases, we used surfaces to highlight certain structural elements of the PT-tracks. To do this, we first created a time-projection of the endogenous T cell signal across all z-planes imaged. This allowed us to visualize the full volume of all the paths traveled by the T cells throughout the movie, thus revealing the overall pattern of cell movement across it. An automated surface was then created using Imaris. In some cases, we also included an additional surface to highlight the lumen of the PT-tracks. For this, counters were manually drawn for each slice in the z stack delineating internal regions, where migration paths were absent. To help identify these areas, time-projection of endogenous T cells were generated. In few movies, seed point surfaces were generated around T cells.

Directionality of cell movement toward or away from the PT-tracks was defined in a binary manner, comparing the progress of the cells in relation to the T zone from their start and end point. We avoided analyzing branching points between PT-tracks because in these regions cell behavior was complex and because high density of cells at these sites limited the number of reliably generated tracks.

Changes in density of endogenous T cells associated with PT-tracks over time were determined by following individual PT-tracks before and after control or LPS treatment. Snap shots were used to define areas of identical size and volume (based on time 0) and the number of cells within them was determined using the spot generating function of Imaris. Density was calculated per mm^3^. To determine the percentage of transferred GFP^+^ T cells associated with PT-tracks after LPS treatment, the number of transferred cells that associated with photoconverted PT-tracks was determined and plotted relatively to the number of cells found in the red pulp at that time point.

#### Motion Sensing Superpixels analysis (MOSES)

To analyze the directionality of collective cellular movement, we took advantage of a recently developed Motion Sensing Superpixels (MOSES) analysis method (Zhou et al., 2019). Individual vessel branch regions and the T zone area were manually segmented. The major and minor axes (as normalized 2d vectors) of each vessel branch were then determined as the eigenvectors corresponding to the larger and smaller eigenvalues of the image moment matrix respectively. Orientation of the computed axes were checked and corrected where applicable by multiplying by −1 to ensure that the major axis of all vessel branches pointed toward the T zone center and all minor axis adopted the same relative orientation relative to the corresponding major axis. Cell motion patterns for each colored cell were described using motion trajectories extracted by MOtion SEnsing Superpixels (Zhou et al., 2019). The image was partitioned into ∼10,000 equal-size square regions of interest or superpixels whose motion was then tracked using dense optical flow to reveal the underlying cellular motion patterns over time. The mean velocity vector and the mean position of the trajectory were used to characterize the average motion of each superpixel over the video duration. A superpixel moves on average toward the T zone if the projected velocity component of its mean velocity vector along the vessel branch it lies within is > 0 (0 = no directionality, < 0 suggests opposite movement). The fraction of vectors that on average move toward the T zone is reported as the number of vectors within vessel regions with positive velocity component toward the T zone divided by the total number of vectors that lie within a vessel. As such, for average motion with no directionality the expected fraction is ∼0.5.

### Quantification and Statistical Analysis

**S**tatistical parameters including number of mice and number of replicates are described in figure legends. The lines represent the means and the error bars represent the standard deviation except for Figures 1C and 2B where it represents the standard error of the mean. Statistical analysis was performed using Prism 7 (GraphPad). When possible a d’Agostino-Pearson nomality test was performed followed by T-Tests or non-parametric Mann -Whitney tests when the distribution was not normal (Figures 5F, 5G, and S4B). For group comparisons, Ordinary One-way ANOVA tests with multiple comparisons were performed except for Figure S2D where we performed Kruskall Wallis tests. P value were calculated and represented as follow: ^∗^(p < 0.5); ^∗∗^(p < 0.01); ^∗∗∗^(p < 0.001); ^∗∗∗∗^(p < 0.0001).

## Supplemental Information

Document S1. Figures S1–S6

Document S2. Article plus Supplemental Information

## References

[bib1] Allen C.D., Okada T., Tang H.L., Cyster J.G. (2007). Imaging of germinal center selection events during affinity maturation. Science.

[bib2] Arnon T.I., Horton R.M., Grigorova I.L., Cyster J.G. (2013). Visualization of splenic marginal zone B-cell shuttling and follicular B-cell egress. Nature.

[bib3] Bajenoff M., Glaichenhaus N., Germain R.N. (2008). Fibroblastic reticular cells guide T lymphocyte entry into and migration within the splenic T cell zone. J. Immunol.

[bib4] Benedict C.A., De Trez C., Schneider K., Ha S., Patterson G., Ware C.F. (2006). Specific remodeling of splenic architecture by cytomegalovirus. PLoS Pathog..

[bib5] Boissonnas A., Fetler L., Zeelenberg I.S., Hugues S., Amigorena S. (2007). In vivo imaging of cytotoxic T cell infiltration and elimination of a solid tumor. J. Exp. Med..

[bib6] Bovetti S., Hsieh Y.C., Bovolin P., Perroteau I., Kazunori T., Puche A.C. (2007). Blood vessels form a scaffold for neuroblast migration in the adult olfactory bulb. J. Neurosci..

[bib7] Brelińska R., Pilgrim C., Reisert I. (1984). Pathways of lymphocyte migration within the periarterial lymphoid sheath of rat spleen. Cell Tissue Res..

[bib8] Butcher E.C., Picker L.J. (1996). Lymphocyte homing and homeostasis. Science.

[bib9] Calderón L., Boehm T. (2011). Three chemokine receptors cooperatively regulate homing of hematopoietic progenitors to the embryonic mouse thymus. Proc. Natl. Acad. Sci. USA.

[bib10] Cheng H.W., Onder L., Novkovic M., Soneson C., Lütge M., Pikor N., Scandella E., Robinson M.D., Miyazaki J.I., Tersteegen A. (2019). Origin and differentiation trajectories of fibroblastic reticular cells in the splenic white pulp. Nat. Commun..

[bib11] Cinamon G., Zachariah M.A., Lam O.M., Foss F.W., Cyster J.G. (2008). Follicular shuttling of marginal zone B cells facilitates antigen transport. Nat. Immunol..

[bib12] Cyster J.G. (1999). Chemokines and cell migration in secondary lymphoid organs. Science.

[bib13] Cyster J.G., Goodnow C.C. (1995). Pertussis toxin inhibits migration of B and T lymphocytes into splenic white pulp cords. J. Exp. Med..

[bib14] Dupré L., Houmadi R., Tang C., Rey-Barroso J. (2015). T Lymphocyte Migration: An Action Movie Starring the Actin and Associated Actors. Front. Immunol..

[bib15] Ford W.L., Gowans J.L. (1969). The traffic of lymphocytes. Semin. Hematol..

[bib16] Förster R., Schubel A., Breitfeld D., Kremmer E., Renner-Müller I., Wolf E., Lipp M. (1999). CCR7 coordinates the primary immune response by establishing functional microenvironments in secondary lymphoid organs. Cell.

[bib17] Girard J.P., Moussion C., Förster R. (2012). HEVs, lymphatics and homeostatic immune cell trafficking in lymph nodes. Nat. Rev. Immunol..

[bib18] Hons M., Kopf A., Hauschild R., Leithner A., Gaertner F., Abe J., Renkawitz J., Stein J.V., Sixt M. (2018). Chemokines and integrins independently tune actin flow and substrate friction during intranodal migration of T cells. Nat. Immunol..

[bib19] Hu J.K., Kagari T., Clingan J.M., Matloubian M. (2011). Expression of chemokine receptor CXCR3 on T cells affects the balance between effector and memory CD8 T-cell generation. Proc. Natl. Acad. Sci. USA.

[bib20] Kaneider N.C., Leger A.J., Kuliopulos A. (2006). Therapeutic targeting of molecules involved in leukocyte-endothelial cell interactions. FEBS J..

[bib21] Khanna K.M., McNamara J.T., Lefrançois L. (2007). In situ imaging of the endogenous CD8 T cell response to infection. Science.

[bib22] Kotani M., Matsuno K., Ezaki T. (1986). Marginal zone bridging channels as a pathway for migrating macrophages from the red towards the white pulp in the rat spleen. Acta Anat. (Basel).

[bib23] Kurachi M., Kurachi J., Suenaga F., Tsukui T., Abe J., Ueha S., Tomura M., Sugihara K., Takamura S., Kakimi K., Matsushima K. (2011). Chemokine receptor CXCR3 facilitates CD8(+) T cell differentiation into short-lived effector cells leading to memory degeneration. J. Exp. Med..

[bib24] Lewis S.M., Williams A., Eisenbarth S.C. (2019). Structure and function of the immune system in the spleen. Sci. Immunol..

[bib25] Lo C.G., Lu T.T., Cyster J.G. (2003). Integrin-dependence of lymphocyte entry into the splenic white pulp. J. Exp. Med..

[bib26] Lu T.T., Cyster J.G. (2002). Integrin-mediated long-term B cell retention in the splenic marginal zone. Science.

[bib27] Luster A.D., Alon R., von Andrian U.H. (2005). Immune cell migration in inflammation: present and future therapeutic targets. Nat. Immunol..

[bib28] Manevich-Mendelson E., Grabovsky V., Feigelson S.W., Cinamon G., Gore Y., Goverse G., Monkley S.J., Margalit R., Melamed D., Mebius R.E. (2010). Talin1 is required for integrin-dependent B lymphocyte homing to lymph nodes and the bone marrow but not for follicular B-cell maturation in the spleen. Blood.

[bib29] Martin F., Kearney J.F. (2000). Positive selection from newly formed to marginal zone B cells depends on the rate of clonal production, CD19, and btk. Immunity.

[bib30] McDole J.R., Wheeler L.W., McDonald K.G., Wang B., Konjufca V., Knoop K.A., Newberry R.D., Miller M.J. (2012). Goblet cells deliver luminal antigen to CD103+ dendritic cells in the small intestine. Nature.

[bib31] Mebius R.E., Kraal G. (2005). Structure and function of the spleen. Nat. Rev. Immunol..

[bib32] Mempel T.R., Scimone M.L., Mora J.R., von Andrian U.H. (2004). In vivo imaging of leukocyte trafficking in blood vessels and tissues. Curr. Opin. Immunol..

[bib33] Mitchell J. (1973). Lymphocyte circulation in the spleen. Marginal zone bridging channels and their possible role in cell traffic. Immunology.

[bib34] Mueller S.N., Hosiawa-Meagher K.A., Konieczny B.T., Sullivan B.M., Bachmann M.F., Locksley R.M., Ahmed R., Matloubian M. (2007). Regulation of homeostatic chemokine expression and cell trafficking during immune responses. Science.

[bib35] Nieuwenhuis P., Ford W.L. (1976). Comparative migration of B- and T-Lymphocytes in the rat spleen and lymph nodes. Cell. Immunol..

[bib36] Nolte M.A., Hamann A., Kraal G., Mebius R.E. (2002). The strict regulation of lymphocyte migration to splenic white pulp does not involve common homing receptors. Immunology.

[bib37] Nolte M.A., Beliën J.A., Schadee-Eestermans I., Jansen W., Unger W.W., van Rooijen N., Kraal G., Mebius R.E. (2003). A conduit system distributes chemokines and small blood-borne molecules through the splenic white pulp. J. Exp. Med..

[bib38] Okada T., Ngo V.N., Ekland E.H., Förster R., Lipp M., Littman D.R., Cyster J.G. (2002). Chemokine requirements for B cell entry to lymph nodes and Peyer’s patches. J. Exp. Med..

[bib39] Ozerlat I. (2011). Multiple sclerosis: Natalizumab improves neurological function in MS. Nat. Rev. Neurol..

[bib40] Saghatelyan A. (2009). Role of blood vessels in the neuronal migration. Semin. Cell Dev. Biol..

[bib41] Schönig K., Schwenk F., Rajewsky K., Bujard H. (2002). Stringent doxycycline dependent control of CRE recombinase in vivo. Nucleic Acids Res..

[bib42] Segarra M., Kirchmaier B.C., Acker-Palmer A. (2015). A vascular perspective on neuronal migration. Mech. Dev..

[bib43] Shah S., Grotenbreg G.M., Rivera A., Yap G.S. (2015). An extrafollicular pathway for the generation of effector CD8(+) T cells driven by the proinflammatory cytokine, IL-12. eLife.

[bib44] Sixt M., Bauer M., Lämmermann T., Fässler R. (2006). Beta1 integrins: zip codes and signaling relay for blood cells. Curr. Opin. Cell Biol..

[bib45] Sörensen I., Adams R.H., Gossler A. (2009). DLL1-mediated Notch activation regulates endothelial identity in mouse fetal arteries. Blood.

[bib46] Spangrude G.J., Sacchi F., Hill H.R., Van Epps D.E., Daynes R.A. (1985). Inhibition of lymphocyte and neutrophil chemotaxis by pertussis toxin. J. Immunol.

[bib47] Sparwasser T., Eberl G. (2007). BAC to immunology--bacterial artificial chromosome-mediated transgenesis for targeting of immune cells. Immunology.

[bib48] Tadayon S., Dunkel J., Takeda A., Halle O., Karikoski M., Gerke H., Rantakari P., Virtakoivu R., Pabst O., Salmi M. (2019). Clever-1 contributes to lymphocyte entry into the spleen via the red pulp. Sci. Immunol..

[bib49] Veiga-Fernandes H., Coles M.C., Foster K.E., Patel A., Williams A., Natarajan D., Barlow A., Pachnis V., Kioussis D. (2007). Tyrosine kinase receptor RET is a key regulator of Peyer’s patch organogenesis. Nature.

[bib50] Victora G.D., Schwickert T.A., Fooksman D.R., Kamphorst A.O., Meyer-Hermann M., Dustin M.L., Nussenzweig M.C. (2010). Germinal center dynamics revealed by multiphoton microscopy with a photoactivatable fluorescent reporter. Cell.

[bib51] von Andrian U.H., Mempel T.R. (2003). Homing and cellular traffic in lymph nodes. Nat. Rev. Immunol..

[bib52] Zhou F.Y., Ruiz-Puig C., Owen R.P., White M.J., Rittscher J., Lu X. (2019). Motion sensing superpixels (MOSES) is a systematic computational framework to quantify and discover cellular motion phenotypes. eLife.

