# Supplementary material for: Visualization of T Cell Migration in the Spleen Reveals a Network of Perivascular Pathways that Guide Entry into T Zones
Source: Immunity. 2020 May 19;52(5):794–807.e7. doi: 10.1016/j.immuni.2020.03.010 (PMC7237890; doi:10.1016/j.immuni.2020.03.010)
Supplement: Document S1. Figures S1–S6 [file mmc1.pdf]

Immunity, Volume *52*

Supplemental Information

Visualization of T Cell Migration in the Spleen Reveals a Network of Perivascular Pathways that Guide Entry into T Zones

Anne Chauveau, Gabriela Pirgova, Hung-Wei Cheng, Angelina De Martin, Felix Y. Zhou, Sarah Wideman, Jens Rittscher, Burkhard Ludewig, and Tal I. Arnon

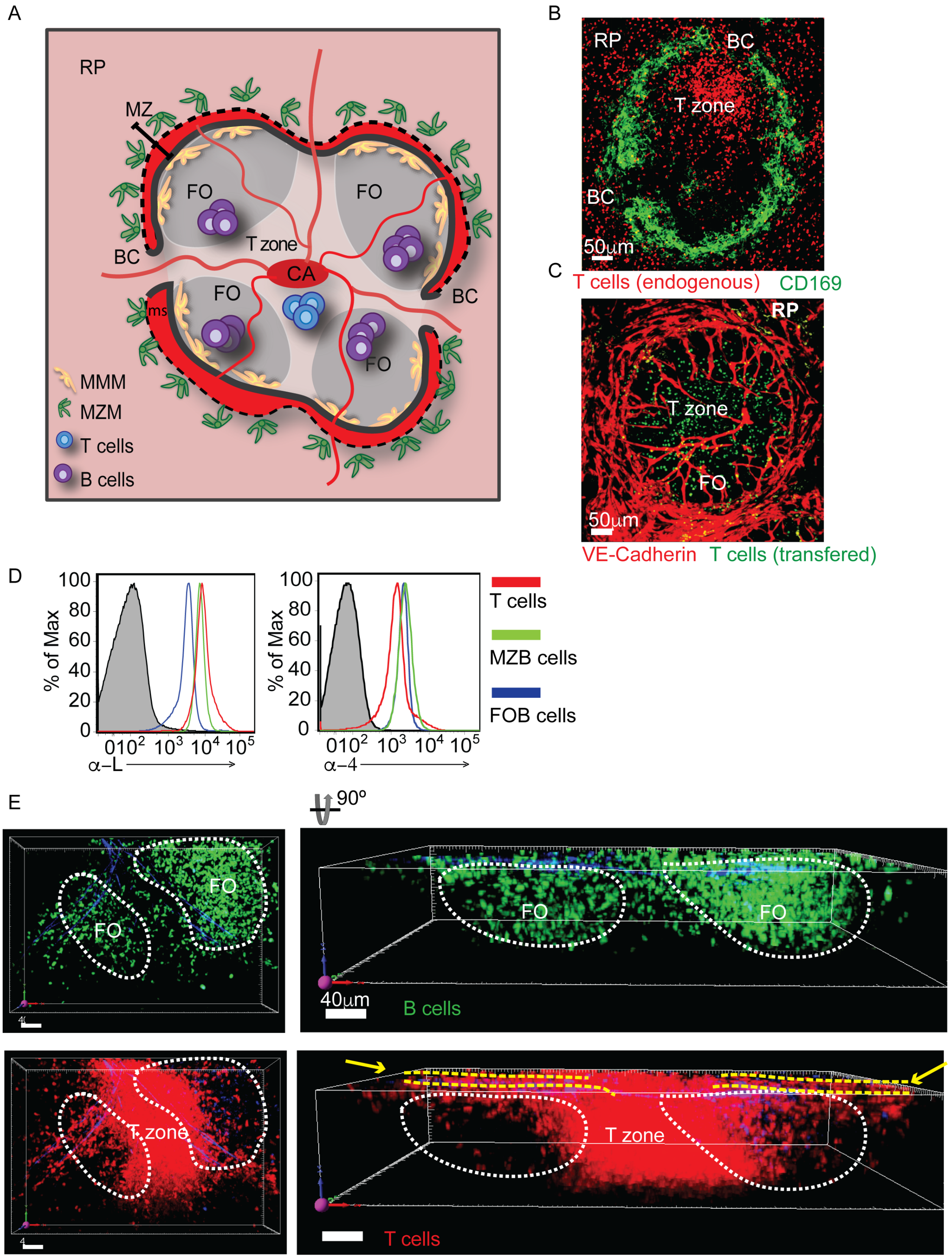

A
RP
MZ
FO
FO
T zone
BC
CA
FO
ms
FO
BC
MMM
MZM
T cells
B cells
B
RP
BC
T zone
BC
50μm
T cells (endogenous)
CD169
C
RP
T zone
FO
50μm
VE-Cadherin
T cells (transfered)
D
100
80
60
40
20
0
% of Max
0 10^2 10^3 10^4 10^5
α-L
α-4
T cells
MZB cells
FOB cells
E
90º
FO
FO
40μm
B cells
T zone
T cells

Figure S1 related to Figure 1. Illustration of the splenic architecture.

(A) Cartoon representing the architecture of a splenic unit. The B cell follicles (FO) and T zone are segregated from the red pulp (RP) by a layer of endothelial cells that lines the marginal sinus. Along these sinuses and around the B cell FO is a narrow compartment known as the marginal zone (MZ). The location of two MZ bridging channels (BC) is represented. Current models identify these sites are areas where the MZ shell is broken and where the T zone directly contacts the MZ and RP. Blood arrives from the circulation and enters the spleen via the central arteriole (CA) that passes through the splenic unit and splits into radial arterioles eventually terminating at the MZ and RP. In these locations, the blood is released allowing circulating cells to passively enter the splenic tissue. To migrate into the white pulp, T cells must engage Gi coupled-protein receptors and cross the marginal sinus layer. Currently, no paths of T cells have been identified within the RP and the model hypothesizes that cells 'drift' from their initial release sites to a BC by the blood flow. Egress of T cells from T zones into the RP is also hypothesized to be mediated via the sites, allowing bi-directional migration of through the BCs. MMM, metallophilic macrophages, MZM, MZ macrophages. (B) 150-μm z-projection view of a fixed hCD2-*DsRed* splenic section stained for CD169 (green). The BCs are identified as areas where the $CD169^+$ metallophilic macrophage (MMM) ring is broken and where the T zone and RP appear connected passing between FO. **(C)** TPLSM of mice expressing tdTomato under the VE-cadherin promoter (red) transferred with CFSE labelled T cells. The circular splenic unit delineated by the vasculature is shown**.** Data in B-C represents at least 2 independent experiments and 5 sections per experiments. (D) Flow-cytometric analysis of the indicated integrin subunits of LFA-1 (α-L) and VLA-4 (α-4) on T cells, MZ and FO B cells. Grey, unstained control. Representative example out of four mice. (E) Z-projection (69-μm) from a movie of a live hCD2-*DsRed* spleen transferred with GFP^+ B cells and imaged 24h later. Tracks of T cells are often associated with collagen bundles (see also Fig 3B), here highlighted by second harmonic (blue). On the top, follicular B cells (green). Bottom, endogenous T cells (red). White dotted lines delineate the boundaries of two B cell follicular regions. Two T cell tracks, pointed at by yellow arrows and highlighted by yellow dotted lines, are shown passing above, but not through, B cell follicles. Images are displayed in two orientations to allow 3D visualization of the relative positioning of the T cell tracks in relation to the T zone and FO (also shown in movie S3). Schematic of the rotation exerted on the initial volume and angle of rotation is shown above each field of view.

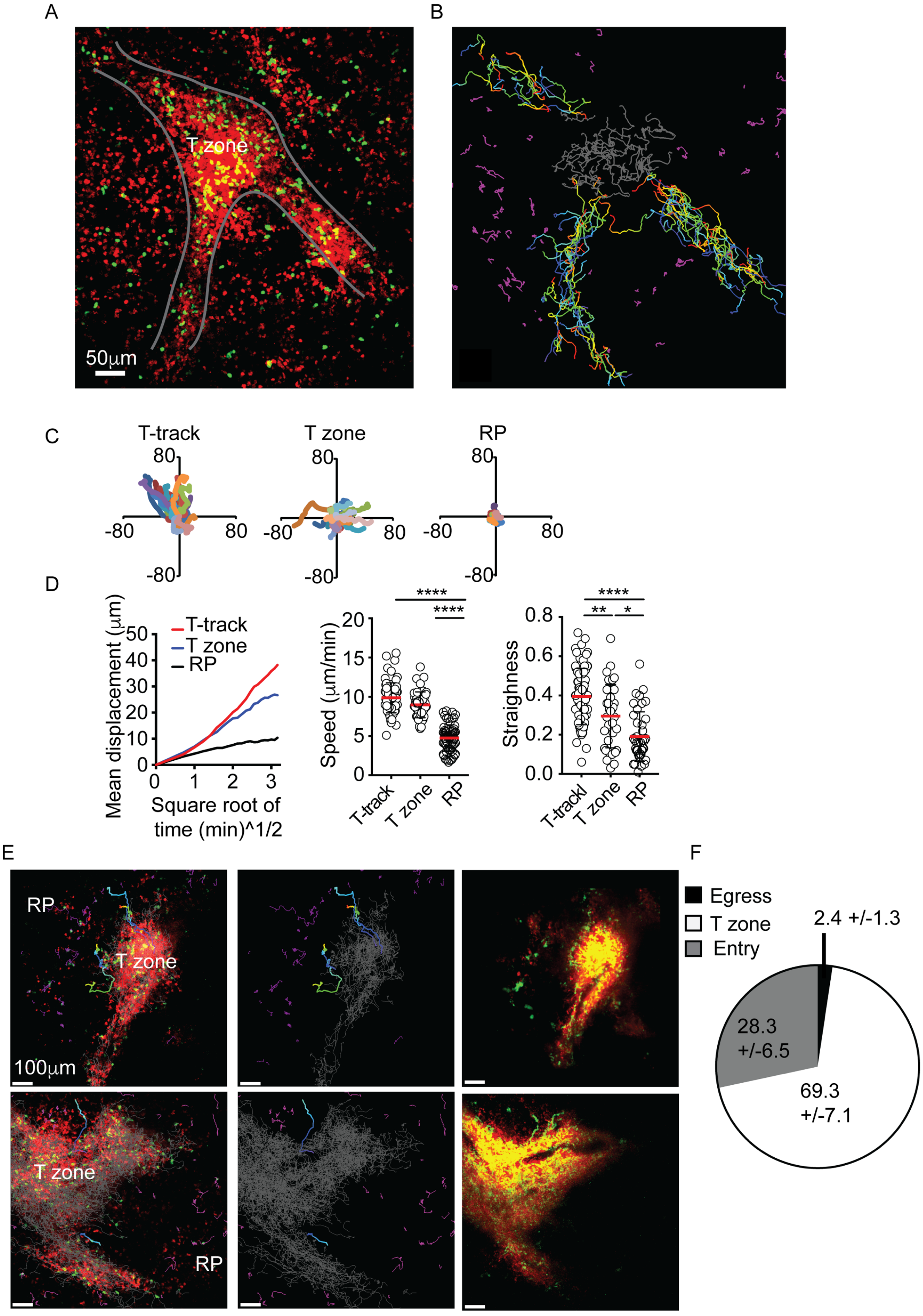

A
B
T zone
50μm
C
T-track
T zone
RP
80
-80
D
Mean displacement (μm)
T-track
T zone
RP
Square root of time (min)^1/2
Speed (μm/min)
Straighness
****
**
*
E
RP
T zone
100μm
F
Egress
T zone
Entry
2.4 +/-1.3
28.3 +/-6.5
69.3 +/-7.1

Figure S2 related to Figure 2. Visualization of T cells migration after short- and long-term transfer.
(A) 81-μm z-projection view from TPLSM intravital imaging of hCD2-*DsRed* mice (endogenous T cells, red) imaged shortly (within 4 hours) after transfer of purified GFP^+ T cells (green). (B) Representative classification of GFP^+ T cell trajectories in the T zone (grey), red pulp (purple) and T cell tracks (time-mapped). (**C**) Superimposed 10-min of randomly selected T cell trajectories in the indicated compartments. (D) Mean displacement versus square root of time, mean velocities and straightness of migration path of transferred GFP^+ T cells in the indicated regions. Data in D represents cells tracked in one representative mouse. Figure shows data from at least 3 independent experiments (E) Left, 81-μm z-projection view from TPLSM intravital imaging of hCD2-*DsRed* mice (endogenous T cells, red) imaged 3 days after transfer of purified GFP^+ T cells (green). Middle, GFP^+ T cell trajectories. Trajectories of cells displaying directional movement away from the T zone are highlighted as 'time-mapped'. Trajectories of cells migrating inside the T zone and on T cell tracks or in the red pulp are in grey and purple, respectively. Image on the left shows time-projection of a single z-plane focusing on deep imaged regions. (F) Pie chart displaying frequencies of cells in the different compartments as outlined above. Data in E and represents cells tracked in one representative mouse out of 3 imaged. Data in F shows the summary of 680 cells collected from 6 movies and 3 mice.

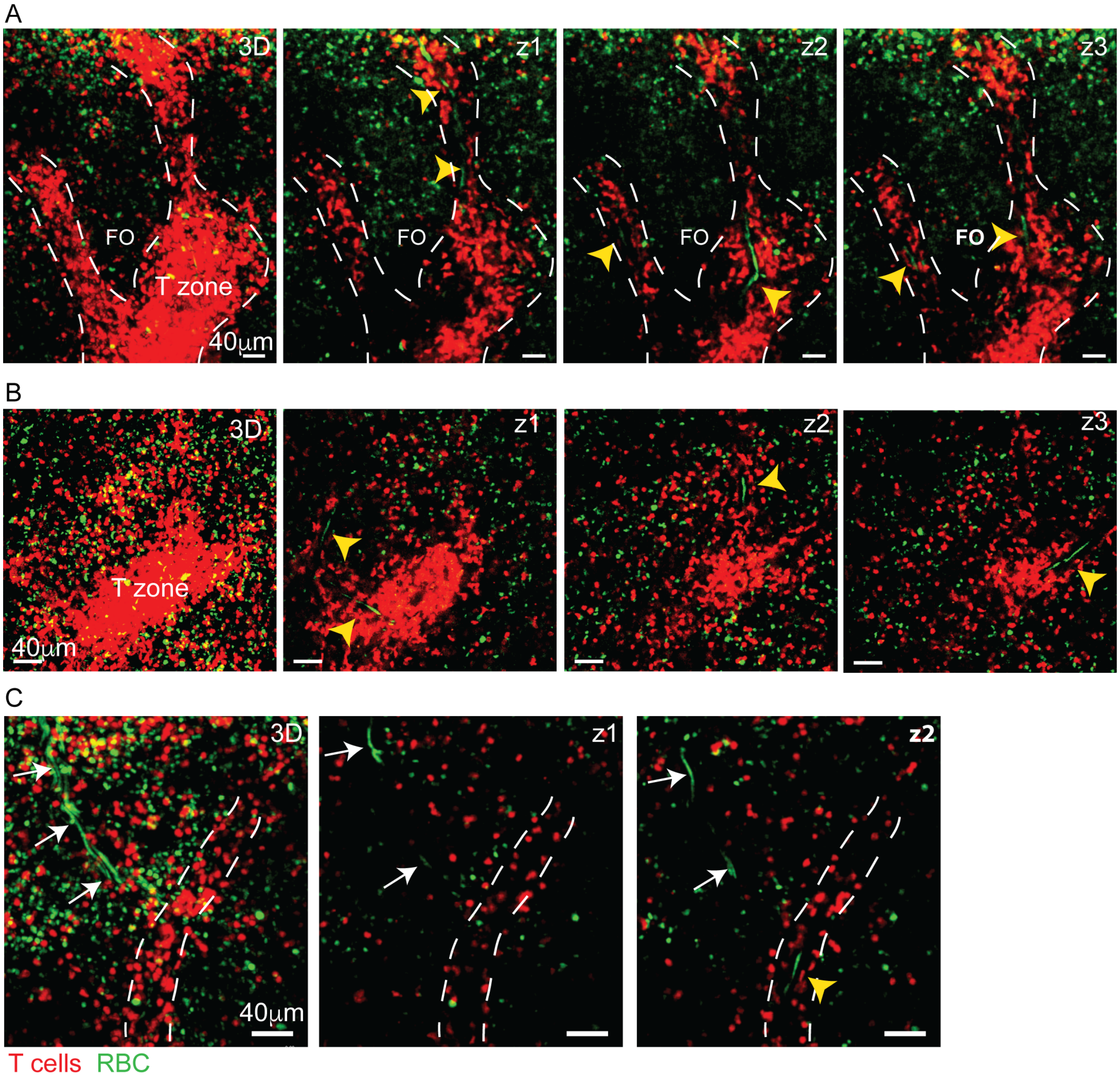

Figure S3 related to Figure 3. Homing paths of T cells are guided by blood vessels.
Three examples highlighting the association of T cell tracks with blood vessels. GFP^{+} red blood cells (RBC, green) were purified and transferred into the tail vein of hCD2-*DsRed* mice minutes before imaging. Snap shots are shown, capturing fast movement of RBC in the blood stream. In each example, the left image shows a 3D projection, to visualize the overall architecture. On the right, focus on different Z plans, where blood flow can be seen as stretched thin lines of green cells, indicating the presence of RBC in transition (also see Movie S6). T cell tracks are marked with white dotted lines. Yellow arrow heads point to RBC flow inside a T cell track. In **C,** an example of a blood vessel that is not associated with a T cell track (pointed to by white arrows) is shown passing next to a T cell track. Data are representative of at least 6 mice and 14 movies acquired.

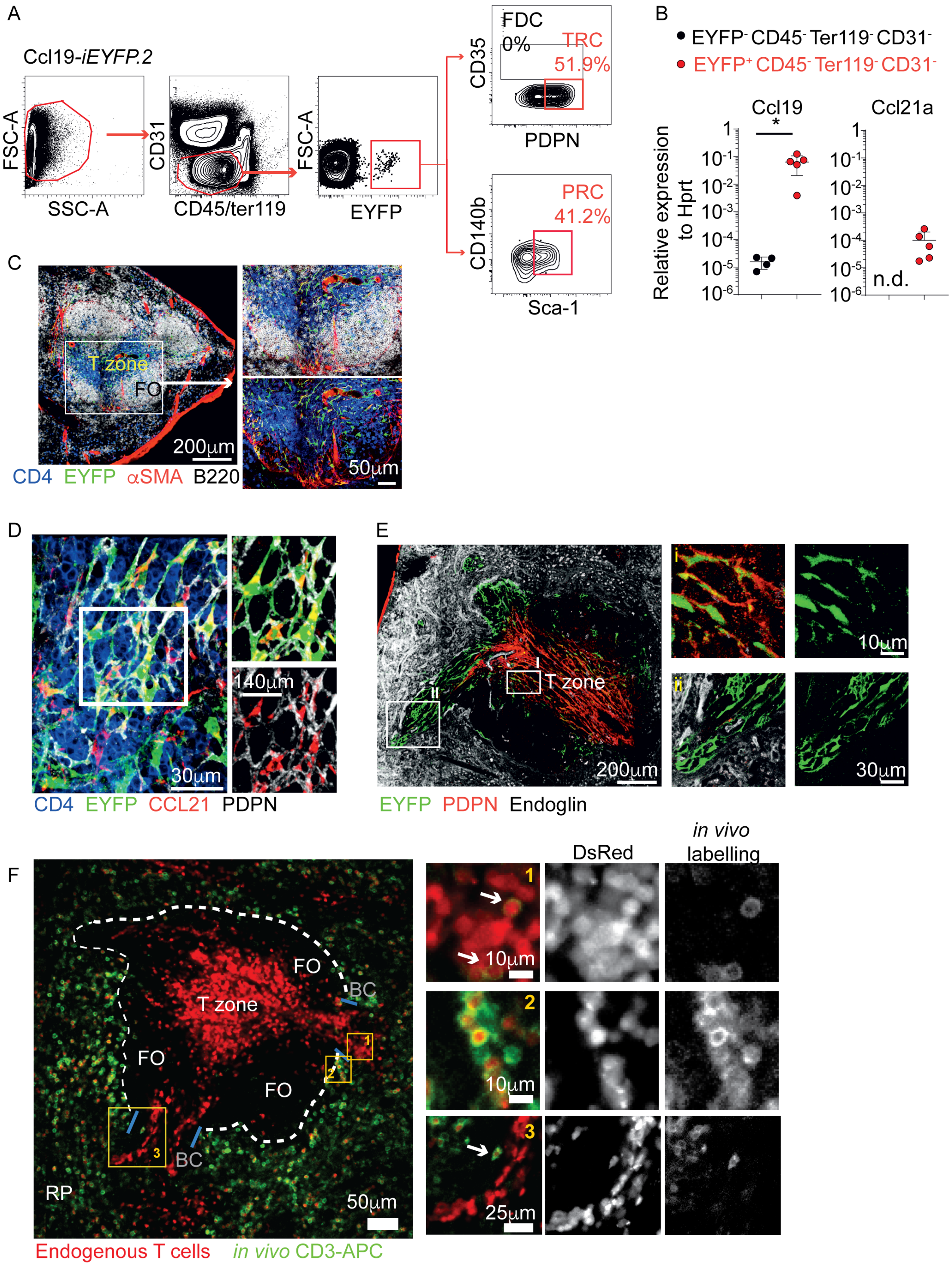

A
Ccl19-iEYFP.2
FSC-A
SSC-A
CD31
CD45/ter119
FSC-A
EYFP
FDC
0%
TRC
51.9%
CD35
PDPN
PRC
41.2%
CD140b
Sca-1
B
EYFP- CD45- Ter119- CD31-
EYFP+ CD45- Ter119- CD31-
Ccl19
Ccl21a
Relative expression to Hprt
*
n.d.
C
T zone
FO
200μm
50μm
CD4 EYFP αSMA B220
D
140μm
30μm
CD4 EYFP CCL21 PDPN
E
T zone
200μm
10μm
30μm
EYFP PDPN Endoglin
F
FO
BC
T zone
FO
FO
BC
RP
50μm
DsRed
in vivo labelling
10μm
10μm
25μm
Endogenous T cells in vivo CD3-APC

Figure S4 related to Figure 4. Genetic targeting of T cell zone reticular cells in the spleen.
Characterization of EYFP expressing cells in Ccl19-*EYFP.2* mice. (A) Representative flow cytometric analysis of viable CD45 and TER119-depleted cells from Ccl19-*iEYFP.2* spleens and analyzed for the indicated markers. Values indicate percentage of the respective population. (B) Quantitative RT-PCR analysis of Ccl19 and Ccl21a expression in sorted $CD45^-TER119^-CD31^-EYFP^+$ and $EYFP^-$ splenic fibroblasts from Ccl19-*iEYFP.2* mouse model. n.d. indicated not detectable (relative expression < 10^{-7}). This analysis shows that compared with the Ccl19-*EYFP* variant that was recently published (Cheng 2019, PMID:30988302), in the Ccl19-*iEYFP.2* model Cre recombinase expression is expressed in a more restricted manner primarily in fibroblastic stromal cell subsets in the T cell zone including perivascular reticular cells (PRC) and T cell zone reticular cells (TRC). Note that the analysis in A and B provides a general characterization of the mice and refers to the total population of EYFP expressing cells in the spleen. However, it does not distinguish between $EYFP^+$ associated channels and $EYFP^+$ cells in the T zone or other sites. (C-E) High-resolution confocal microscopy of frozen spleen sections from adult Ccl19-*EYFP.2* mice stained with the indicated antibodies. Boxes show magnified areas on the right. Image in (C) illustrates the overall EYFP expression pattern in the spleen in relation to the B cell follicles (FO) and T zone. (D) Focus on a T zone showing $EYFP^+$ reticular cells co-expressing CCL21 and podoplanin (PDPN). (E) Stained for $EYFP^+$ reticular cells co-expressing PDPN and Endoglin. (F) An example of hCD2-*DsRed* fixed spleen sections after *in vivo* labeling for 5 min by intravenous injection of anti CD3-APC. Endogenous T cells, red. APC signal, green. White dotted line highlights the MZ-FO border. On the right, magnified areas focusing on PT-track associated cells (box 1,3) or on cells in the MZ (box 2). White arrows (magnified box 1 and 3) point to few labeled cells that are associated with PT-tracks. Data are representative of 3 independent experiments.

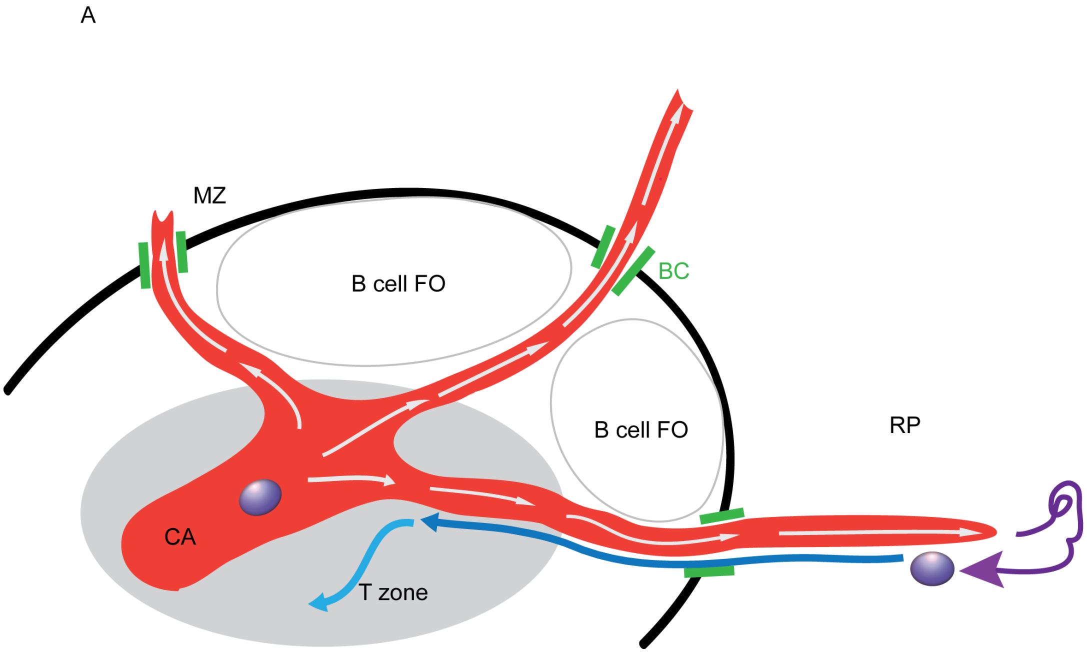

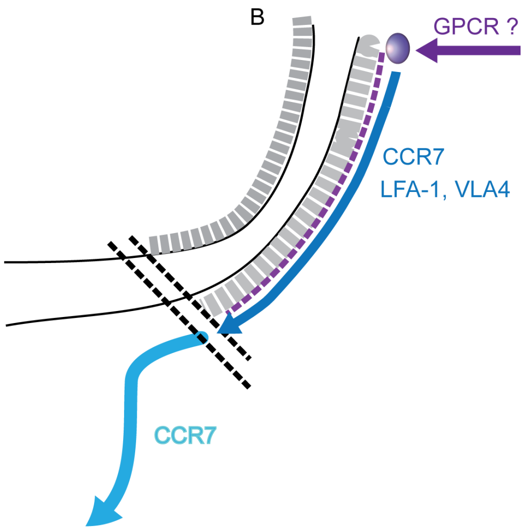

Figure S5 related to Figure 6.: A revised model for T cell entry into the splenic T zones.
Circulating naïve T cells enter the spleen from the blood by passing through central arterioles (CA). Inside the blood vessels, T cells move passively with the flow (grey arrows). The CA splits into smaller vessels that open and release their blood content at the marginal zone (MZ) and red pulp (RP). From these sites, T cells backtrack their steps following the outer layer of the blood vessels directly into T zones. Having described these paths, we refer to them as perivascular T-tracks ('PT-tracks') to distinguish them from other T cell migratory paths that have been previously described in the spleen. Cell attraction to the PT-tracks depend on activation of GPCRs (purple arrow and purple dotted line, bottom). Once attached, T cells migrate in a one-directional manner along the PT-tracks towards the T zone, guided by CCR7 (blue arrows). Movement is regulated by a layer of perivascular stroma cells that coat the blood vessels (bottom, grey dotted line). LFA-1 and VLA-4 contribute to this process by enhancing cell velocities. At the contact point with the MZ, the PT-tracks pass via bridging channels (BC, yellow lines). To complete entry and reach the T zone compartment, the cells eventually detach from the PT-tracks and 'dive' down into the T-zone that lies underneath (detachment point is highlighted by black dotted lines, bottom). This step is CCR7-dependent, as *CCR7*$^{-/-}$ T cells fail to perform this movement and instead accumulate at the outer rim of the T zone.

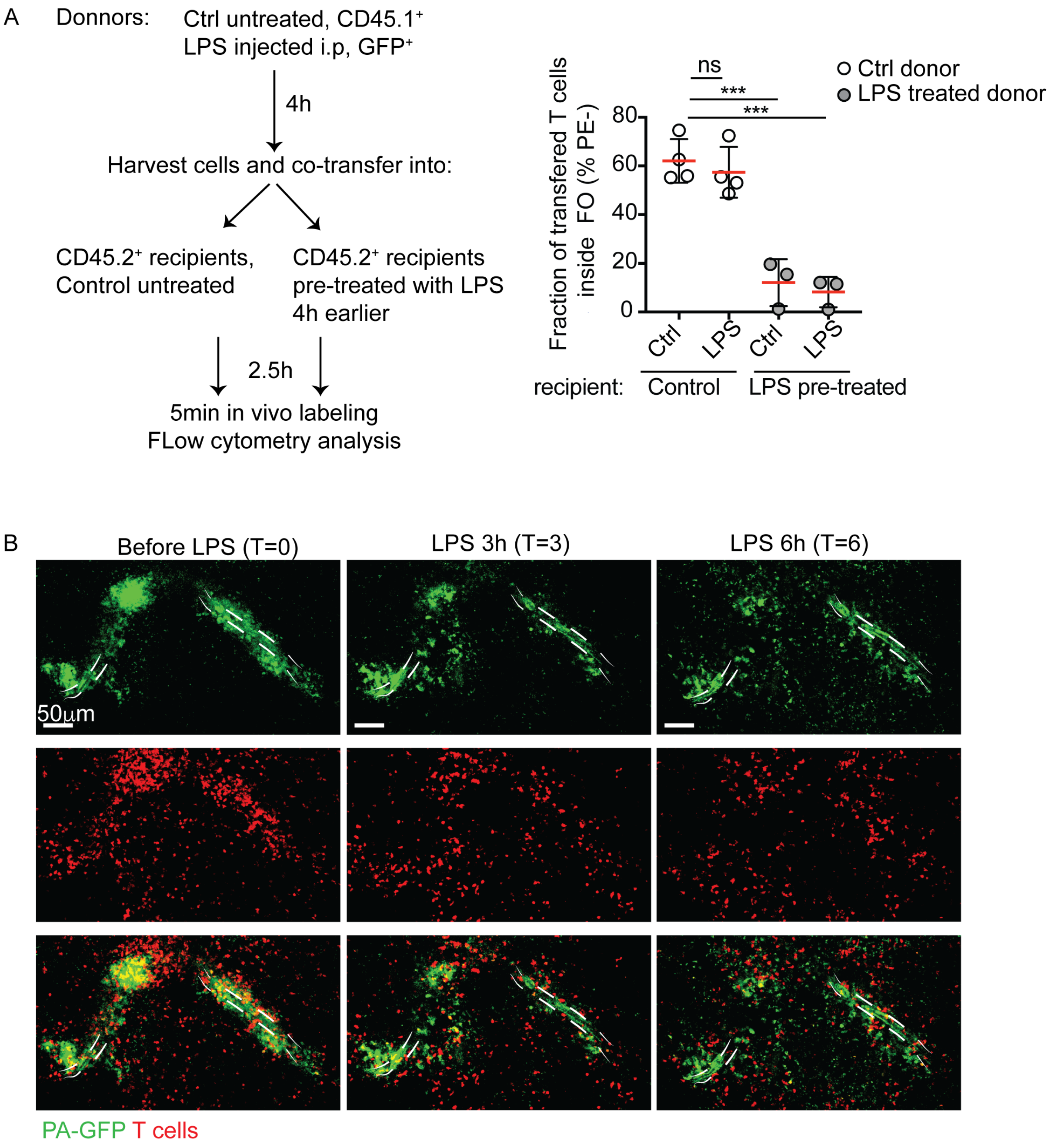

Figure S6 related to Figure 7. During inflammation the PT-tracks are modified, and T cell entry is blocked. (A) Left, schematic illustration of experimental design. GFP$^+$ mice were injected with LPS. Four hours later, cells were harvested and mixed with cells derived from control (untreated) CD45.1$^+$ donors. The mixture was adoptively transferred into CD45.2$^+$ hosts that were either untreated (control) or injected with LPS 4h earlier. Two and a half hours later, mice were *in vivo* labelled with CD3-PE for 5min and entry was assessed by flow cytometry. Right, the frequencies of GFP$^+$ and CD45.1$^+$ T cells that entered the follicles (PE$^-$). Data represent one experiment out of 3 performed. (B) Individual PT-tracks were photoconverted in live Ubi-*PA-GFP*$^{+/-}$ hCD2-*DsRed* $^{+/-}$ mice. Images were collected before and after LPS administration, at the indicated time points. Shown are examples for 2 photoconverted PT-tracks monitored over time. Top, photoconverted cells shown in green. Grey lines highlight 2 photoconverted PT-tracks. Middle, endogenous T cells within the same field of view, red. Bottom, overlap of photoconverted and endogenous T cells. The summary of densities of endogenous T cells measured along each PT-track is shown in Figure 7.
